# Lysophospholipids and branched chain amino acids are associated with aging: a metabolomics-based study of Chinese adults

**DOI:** 10.1186/s40001-023-01021-w

**Published:** 2023-02-02

**Authors:** Yiming Pan, Pan Liu, Shijie Li, Bowen Li, Yun Li, Lina Ma

**Affiliations:** 1 Department of Geriatrics, Xuanwu Hospital, Capital Medical University, National Research Center for Geriatric Medicine, Beijing, 100053 China; 2 LipidALL Technologies Company Limited, Changzhou, 213022 Jiangsu China

**Keywords:** Aging, Metabolomics, Lysophospholipid, Branched-chain amino acid, Biomarker

## Abstract

**Background:**

Aging is an inevitable process associated with impairments in multiple organ systems, which increases the risk of comorbidity and disability, and reduces the health-span. Metabolomics is a powerful tool in aging research, which can reflect the characteristics of aging at the level of terminal metabolism, and may contribute to the exploration of aging mechanisms and the formulation of anti-aging strategies.

**Methods:**

To identify possible biomarkers and pathways associated with aging using untargeted metabolomics methods, we performed liquid chromatography–mass spectrometry (LC–MS)-based untargeted metabolomics profiling on serum samples from 32 older adults and 32 sex-matched young controls.

**Results:**

Metabolite profiling could distinguish the two groups. Among the 349 metabolites identified, 80—including lysophospholipids whose levels gradually decline—are possible candidate aging biomarkers. Valine, leucine and isoleucine degradation and biosynthesis were important pathways in aging, with reduced levels of l-isoleucine (*r* = − 0.30, *p* = 0.017) and l-leucine (*r* = − 0.32, *p = *0.010) observed in older adults.

**Conclusions:**

We preliminarily revealed the metabolite changes associated with aging in Chinese adults. Decreases in mitochondrial membrane-related lysophospholipids and dysfunction of branched-chain amino acid metabolism were determined to be the characteristics and promising research targets for aging.

**Supplementary Information:**

The online version contains supplementary material available at 10.1186/s40001-023-01021-w.

## Background

Aging has been broadly defined as a time-dependent functional decline, accompanied by a gradual loss of physiological capacity and an increased risk of disease or death [1]. Aging is an inevitable natural process associated with multiple pathological and physiological changes at the gene, protein, and molecular levels, and is closely associated with many chronic diseases [1–4]. Global human life expectancy has increased dramatically over the past decades, and predictive models suggest that it will continue to rise [5]. As a consequence, the increasingly aging population greatly challenges the formulation of healthcare policies and socioeconomic development. Delaying functional decline and preventing age-related diseases and disabilities have thus become urgent public health priorities and major scientific issues in geriatrics [6].

It is well-known that chronological age cannot fully reflect physiological function, as the rate at which physiological function declines with time varies greatly among individuals. Compared to chronological age, biomarkers could be more helpful to quantify functional capacity, to identify individuals at risk of age-related diseases, and to monitor and evaluate the effectiveness of interventions [7]. The lack of a comprehensive set of aging biomarkers is an obstacle to aging research. At present, some studies on aging have focused on biomarkers specific to some aging phenotypes, such as Alzheimer's disease [8], heart failure [9] and pulmonary fibrosis [10], while others have explored biomarkers of longevity by concentrating on centenarians [11, 12]. Other preclinical studies have tried to identify the molecular features and mechanisms of aging in animal models, such as mice [13], *Caenorhabditis elegans* [14] and *Drosophila* [15]. The complex mechanism of aging dictates that the aging phenotype requires multiple parameters to be described. However, research on aging biomarkers is not comprehensive and systematic, and age-related changes in the metabolic profiles of humans are largely unclear.

Metabolomics is an emerging technology for the detection of the final metabolites of cells. It can completely reflect the phenotype of an organism under the joint effects of genetics and environment. Compared with other omics platforms, metabolomics has unique advantages in biomarker research. In the present study, we performed untargeted metabolomics analysis on serum samples from older and young adults, aiming to identify biomarkers and pathways related to aging or decline in physical function.

## Methods

### Study design and participants

A total of 64 inpatients who presented for medical examination at Xuanwu Hospital were enrolled in our study, including 32 older adults aged 60 years or older and 32 sex-matched young adults aged between 36 and 59 years. We excluded individuals with malignant tumors, dysfunction of one or more organs, or who were receiving glucocorticoid or immunomodulatory therapy. We collected data related to general characteristics (age, sex, weight, height, smoking, and drinking), physical capacity (like 4-m gait speed and maximum grip strength), blood pressure, medical history, prescribed drugs, and laboratory test results. The body mass index (BMI) was calculated as the weight in kilograms divided by the square of the height in meters. The 4-m gait speed was calculated by dividing 4 m by the seconds it takes to walk this distance on flat ground without assistance. Grip strength was defined as the maximum strength of both hands while standing upright, with arms hanging naturally down. Creatinine clearance (CCr) was estimated using the Cockcroft–Gault equation.

### Serum sample collection

After at least 8 h of overnight fasting, smoking and alcohol abstinence, blood samples from participants were collected and centrifuged. Serum was drawn and stored in cryogenic tubes at − 80 °C within 2 h.

### Metabolomics analysis

#### Reagents

The water was purified using an ultrapure water preparation system. All internal standard references were obtained from Cambridge Isotope Laboratories (USA). LC–MS-grade acetonitrile and methanol were obtained from Merck (Germany). HPLC-grade formic acid was obtained from Sigma (Germany).

#### Metabolome extraction [16]

Briefly, 50 µL of serum was added to 200 µL of ice-cold methanol and incubated with shaking for 30 min at 1500 rpm and 4 °C, followed by centrifugation for 10 min at 12,000 rpm and 4 °C. The supernatant was transferred into a clean 1.5 mL centrifuge tube and dried using SpeedVac. The dried extracts were redissolved in 1% acetonitrile in water containing internal standards, and the upper layer liquids were collected for LC–MS analysis. Quality control (QC) samples were prepared by mixing all serum samples in steps identical to those used for the actual serum samples.

#### Instruments [17]

The column ACQUITY UPLC HSS T3 1.8 µm, 2.1 × 100 mm columns, and ultra-performance liquid chromatography coupled to quadrupole–TOF MS were applied. The temperatures of the column and auto-sampler were maintained at 40 °C and 4 °C, respectively. The injection volume was 5 μL per run Flow rate was 0.35 mL/min. Mobile phase A was water containing 0.1% formic acid (v/v), and mobile phase B was acetonitrile. The following linear gradient was used: 0–1.0 min with 2% B, 1.0–6.0 min with 2–42% B, 6.0–8.0 min with 42–65% B, 8.0–10.0 min with 65–76% B, 10.0–11.0 min with 76–100% B, 11.0–14.0 min with 100–100% B. The MS parameters for detection were: ESI (−) source voltage − 4.5 kV, and + 5.5 kV for ESI (+); vaporizer temperature, 500 °C; drying gas (N2) pressure, 50 psi; nebulizer gas (N2) pressure, 50 psi; curtain gas (N2) pressure, 35 psi; the scan range was *m/z* 60–600. Information-dependent acquisition mode was used for MS/MS analyses of the metabolites. The collision energy was set at 35 ± 15 eV. Data acquisition and processing were performed using Analyst^®^ TF 1.7.1 Software.

#### Data processing

All detected ions were extracted using MarkerView 1.3 in the format of two dimensional matrix, including mass to charge ratio (*m*/*z*), retention time and peak areas, and isotopic peaks were filtered. Raw MS data were extracted with the parameter setting as retention time tolerance less than 0.1 min during alignment. Each metabolites across the whole batch followed this retention time rule, otherwise it would be removed. PeakView 2.2 was applied to extract MS/MS data and to perform a comparison with the Metabolites database, HMDB, METLIN, and standard references to annotate ion ID. The self-compiled R program was used for the statistical analysis.

### Statistical analysis

For the general characteristics and basic clinical data, continuous variables are shown as the mean (standard deviation); categorical variables are shown as frequencies (percentages). Continuous variables were compared using Student’s *t* test, and categorical variables were compared using the Pearson’s χ^2^ test. Pearson correlation analysis was used to describe correlations between metabolites or physical functions and age. Differences were considered to be statistically significant with two-sided *p* value < 0.05.

For metabolomics data, metabolites whose content was below the detection limit were imputed with zero. Principal component analysis (PCA) and orthogonal partial least squares discriminant analysis (OPLS-DA) were performed to distinguish old and young groups and to screen potential aging biomarkers (with variable importance in the projection > 1 and *p* value < 0.05). We performed a random permutation test and computed the area under the receiver operating characteristic (ROC) curve (AUC) on the OPLS-DA model to evaluate it. The peak areas of metabolites were compared using Student’s *t* test. Metabolites with fold change > 1.5 or < 1/1.5 and *p* < 0.05, were more meaningful to aging.

### Pathway analysis

Metabolite set enrichment analysis (MSEA) [18] and metabolic pathway analysis (MetPA) [19] based on SMPDB (https://smpdb.ca) and KEGG (https://www.kegg.jp/) were used to identify over-represented metabolite sets and pathways related to aging. Metabolites with two-sided *p* values < 0.05, as determined by Student’s *t* test, were included in the pathway analysis, and those without matched HMDB ID were excluded. Metabolite sets with fold enrichment > 2 and raw *p* value < 0.05, and pathways with raw *p* value < 0.05, were considered as more significant to aging.

## Results

### Basic information of the participants

The basic characteristics of the 64 sex-matched participants with serum UPLC–QTOF data are summarized in Table1. Older participants tended to be slower (*p* = 0.011) and weaker (*p* < 0.001), had a greater pulse pressure (*p* = 0.003) and more chronic diseases (*p* = 0.001), and took more drugs (*p* < 0.001). Older people showed lower levels of creatinine clearance, albumin, hemoglobin, total cholesterol, and low-density lipoprotein and higher levels of D-dimer. Grip strength (r = − 0.64, *p* < 0.0001) and gait speed (r = − 0.54, *p* < 0.0001) were strongly negatively correlated with age.

**Table 1:** Basic characteristics of young and old groups

Variables		Young (*n* = 32)	Old (*n* = 32)	*p* value
General information	Age (years), mean (SD)	53.59 (5.71)	76.06 (8.64)	< 0.001
	Male, no (%)	24 (75)	24 (75)	1.000
	Weight (Kg), mean (SD)	76.39 (14.67)	69.28 (9.98)	0.027
	Height (cm), mean (SD)	170.47 (8.15)	167.53 (5.97)	0.105
	BMI (Kg/m^2^), mean (SD)	26.17 (4.01)	24.63 (3.03)	0.089
	Smoking, no (%)	11 (34.4)	8 (25.0)	0.412
	Drinking, no (%)	13 (40.6)	6 (18.8)	0.055
Physical capacity	Gait speed (m/s), mean (SD)	1.14 (0.38)	0.84 (0.42)	0.011
	Grip strength (Kg), mean (SD)	38.5 (8.7)	28.5 (8.4)	< 0.001
Blood pressure	SBP (mmHg), mean (SD)	131.97 (16.89)	136.53 (19.19)	0.317
	DBP (mmHg), mean (SD)	80.06 (10.46)	72.97 (12.70)	0.018
	PP (mmHg), mean (SD)	51.91 (13.66)	63.56 (16.06)	0.003
Medical history	Hypertension, no (%)	21 (65.6)	24 (75)	0.412
	Coronary heart disease, no (%)	4 (12.5)	9 (28.1)	0.120
	Chronic obstructive pulmonary disease, no (%)	0 (0.0)	1 (3.1)	0.313
	Cerebravascular accident, no (%)	3 (9.4)	13 (40.6)	0.004
	Type 2 diabetes mellitus, no (%)	13 (40.6)	13 (40.6)	1.000
	Number of chronic diseases, mean (SD)	2.91 (2.29)	5.41 (3.11)	0.001
	Number of drugs, mean (SD)	3.03 (2.21)	5.72 (3.07)	< 0.001
Laboratory tests	Creatinine (µmol/L), mean (SD)	66.50 (21.69)	76.44 (27.27)	0.112
	CCr (mL/(min*1.73 m^2^)), mean (SD)	119.17 (33.88)	75.91 (29.25)	< 0.001
	Alanine aminotransferase (IU/L), mean (SD)	24.53 (12.61)	22.00 (12.55)	0.424
	Aspartate aminotransferase (IU/L), mean (SD)	23.65 (4.77)	24.19 (8.01)	0.746
	Albumin (g/L), mean (SD)	41.35 (2.77)	38.98 (3.73)	0.007
	Hemoglobin (g/L), mean (SD)	146.53 (13.70)	133.69 (14.87)	0.001
	Triglycerides (mmol/L), mean (SD)	2.08 (1.10)	1.53 (1.04)	0.052
	Total cholesterol (mmol/L), mean (SD)	4.51 (0.78)	3.97 (0.86)	0.015
	High-density lipoprotein (mmol/L), mean (SD)	1.07 (0.31)	1.22 (0.40)	0.131
	Low-density lipoprotein (mmol/L), mean (SD)	2.75 (0.69)	2.32 (0.66)	0.015
	C reactive protein (mg/L), mean (SD)	2.83 (2.81)	4.69 (4.97)	0.114
	d-dimer (µg/mL), mean (SD)	0.20 (0.12)	0.59 (0.83)	0.012

*SD* standard deviation, *BMI* body mass index, *SBP*, systolic blood pressure, *DBP* diastolic blood pressure, *PP* pulse pressure, *CCr* creatinine clearance

### Quality of data

The QC samples were clustered well in the PCA score plot with good consistency (Additional file 1: Fig. S1). In the paired comparison matrix of 13 QC samples after log10 conversion, the correlation coefficients were all greater than 0.99 (Additional file 1: Fig. S2). After correction by internal standards, the average relative standard deviation value in QCs was 11.0%, indicating high quality of the data in this work.

### Metabolomics profile of participants

**Fig. 1:**
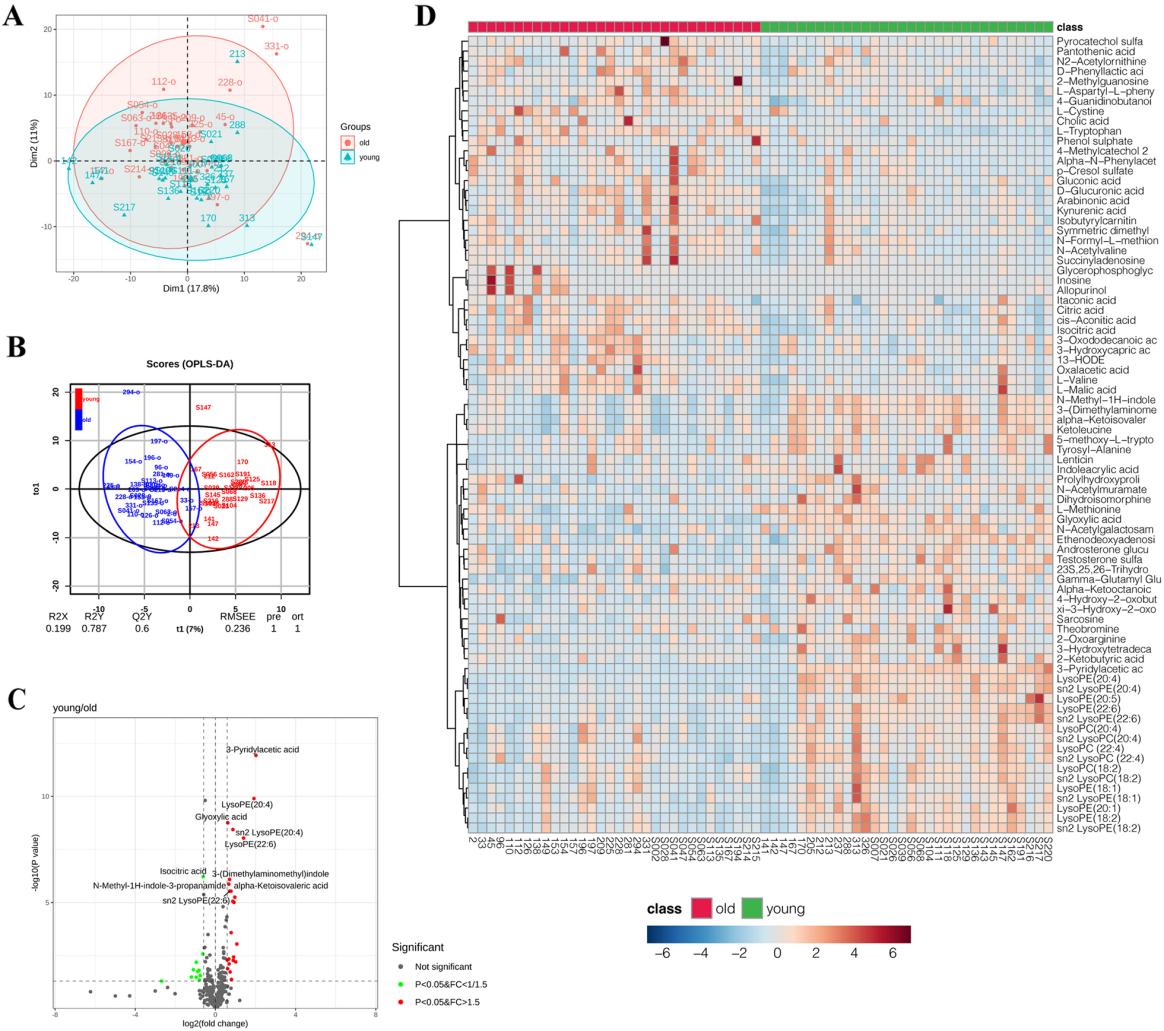
**A** Individual plot of PCA between old and young groups. Samples of two groups show a clear distinction in the PCA of the first two dimensions. The percentage on the X/Y axis refers to the ratio of inter-group differences explained by the first two principal components, respectively. **B** OPLS-DA scores plot of metabolites from old and young groups. **C** Volcano plot of metabolites with FC (fold change) > 1.5 or < 1/1.5, and *p* value < 0.05 in T test. Of them 11 are up-regulated and 23 are down-regulated in older adults. **D** Heatmap of standardized content of 80 differential metabolites from old and young groups. White represents that the average value of metabolite content is 0. The bar below shows the value in color, which means how many standard deviations from the mean value

By comparing with 66 standard compounds (Additional file 1: Table S1), 349 metabolites of 46 classes were identified from serum samples using ultra-performance liquid chromatography (UPLC)–quadrupole time-of-flight (QTOF) mass spectrometry. The class, name, database ID and confidence level [20] of identified metabolites were listed in Additional file 1: Tables S2 and S3. In PCA, the first two principal components (Dimention-1: 17.8%, Dimention-2: 11%) accounted for nearly 30% of the inter-group differences (Additional file 1: Fig. S3Aa–c). The metabolomics data of older adults could be distinguished from those of young individuals in PCA score plot (Fig. 1A). We performed OPLS-DA on the identified metabolites and observed significant separation between the old and young groups (Fig. 1B). The model was proven to work in a random permutation test (Additional file 1: Fig. S3Ba, b), and showed great sensitivity (0.97) and specificity (1.00) in the receiver operating characteristic (ROC) curve (Additional file 1: Fig. S3B, c). There were 76 metabolites with variable importance in the projection (VIP) > 1 and *p* < 0.05, in the *t* test, 34 with fold change (FC) > 1.5 or < 1/1.5 and *p* < 0.05 (Fig. 1C), and 30 with VIP > 1, FC > 1.5 or < 1/1.5, and *p* < 0.05 (Additional file 1: Table S4), respectively, which were considered as candidate biomarkers of aging with statistical differences (Fig. 1D). Of the 80 differential metabolites we identified, we observed lower levels of lysophosphatidylcholine (LPC) and lysophosphatidylethanolamine (LPE), including LysoPC (18:2, 20:4, 22:4), sn2 LysoPC (18:2, 20:4, 22:4), LysoPE (20:1, 20:4, 20:5, 18:1, 18:2, 22:6), and sn2 LysoPE (18:1, 18:2, 20:4, 22:6), in the serum samples of older adults.

### Pathway analysis

**Fig. 2:**
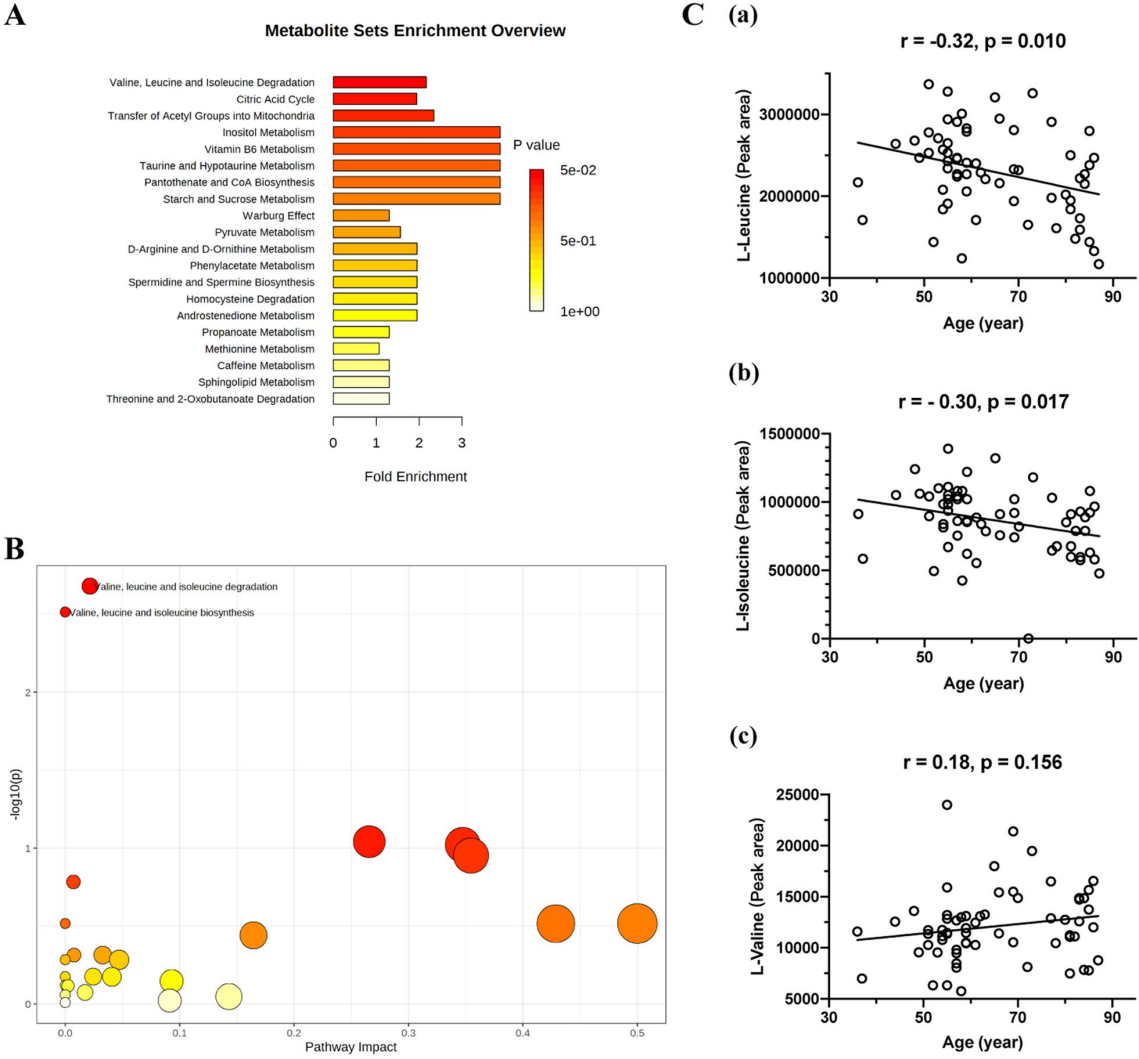
**A** Metabolite sets with fold enrichment > 1 in MSEA (metabolite set enrichment analysis). Metabolites without matching HMDB ID were removed. The *X* axis refers to the fold enrichment of the pathway. The bar on the right side shows p value in color. **B** Pathways with raw p < 0.05 in MetPA (Metabolic pathway analysis). Metabolites without matching HMDB ID were removed. The *X* axis refers to the pathway impact, and *Y* axis refers to − log10(*p*). Larger and redder dot represents more meaningful pathway. **C** Pearson correlation between the peak area of BCAAs (Branched-chain amino acids) and age. l-Leucine and l-Isoleucine show negative association with age, while l-Valine shows no statistical correlation

Among the metabolite sets with fold enrichment > 1 (Fig. 2A), valine, leucine and isoleucine degradation (fold enrichment = 2.16) was the only one with raw *p* < 0.05 in enrichment analysis for aging (with up-regulation of l-valine [Val] and down-regulation of alpha-ketoisovaleric acid, l-isoleucine [Ile], leucine [Leu], and ketoleucine) (Additional file 1: Table S5). The pathways associated with aging were valine, leucine and isoleucine degradation, and valine, leucine and isoleucine biosynthesis (Fig. 2B, Table S6).

### Associations between branched chain amino acids (BCAAs) and age

In the three BCAAs, l-Leu (*r* = − 0.32, *p*-0.010) and l-Ile (*r* = − 0.30, *p* = 0.017) were negatively associated with age, while l-Val had no statistical association with age (Fig. 2C).

## Discussion

**Fig. 3:**
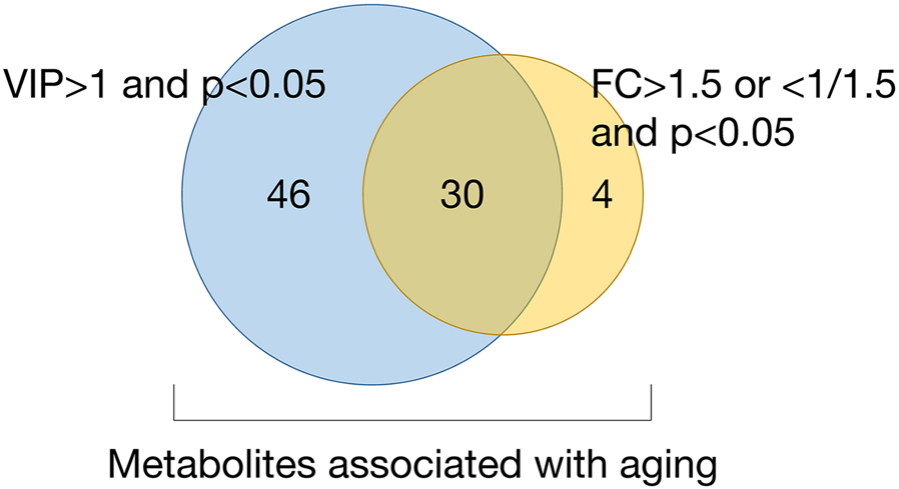
Venn diagram of possible aging-related differential metabolites

**Fig. 4:**
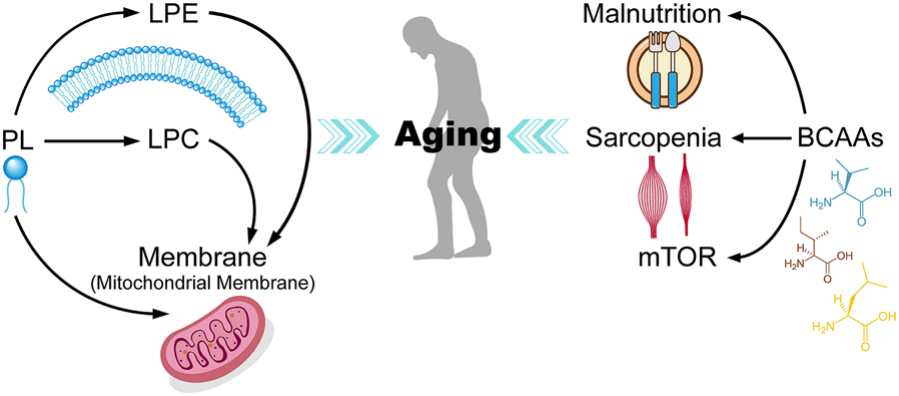
Possible underlying mechanism of aging. Down-regulation of LPEs and LPCs is a feature of aging, which may be related to the synthesis and function of mitochondrial membranes; Dysfunction of BCAA metabolism probably has two-sided effects on aging

This study screened candidate aging biomarkers by untargeted metabolomics analysis and preliminarily explored the mechanisms underlying aging in a Chinese population. The young and old groups showed different clinical characteristics and could be clearly distinguished by their metabolic profiles. In total, 80 metabolites (including six glycerophosphocholines and ten glycerophosphoethanolamines) (Fig. 3) and two pathways (valine, leucine and isoleucine degradation and biosynthesis) were considered to be associated with aging. The relationship between lysophospholipid-related metabolites, BCAA metabolism, and aging will be discussed in detail below (Fig. 4).

### Down-regulation of lysophospholipid (LP) levels are relevant to aging

LPC and LPE are important precursors for the biosynthesis of phospholipids (PLs), including phosphatidylcholine (PC) and phosphatidylethanolamine (PE), which are essential for maintaining the structure and function of biological membranes (including mitochondrial membranes) [21–23]. We observed that serum levels of six LPC and ten LPE were down-regulated in older adults compared to young. In the Baltimore Longitudinal Study of Aging, lower plasma LPC was correlated with impairment of mitochondrial oxidative capacity after adjusting for age, sex, and height [24]. In terms of physical function, lower LysoPC (18:2) could longitudinally predict a greater decrease in gait speed in older adults [25]. A decline in circulating LPs has been associated with many age-related diseases, such as cardiovascular diseases [26], arterial stiffening [27], Alzheimer's disease [28, 29] and several types of cancer [30]. A similar phenomenon exists in aging animal models; in previous studies, PC and PE levels were reported to decrease with age in *Caenorhabditis elegans*, rats, and mice [31]. The longitudinal decline of LPC (18:0) and LPE (20:4, 22:6, 36:2, 36:3, 38:4, 38:5, 38:6, 40:6, and 40:7) with age in mouse kidney tissue was also observed by lipidomics analysis [32]. These data suggest that LPs are closely associated with aging. Quantification of circulating LPs could be a promising method to reflect the degree of aging or to influence LP levels through diet or drugs as potential interventions for aging. However, a systematic review showed that the sex differences in LPCs and PLs in aging are notable; indeed, PC, PE, and LPC decreased with age in men, while LPC and PE increased with age in women [33].

### Dysfunction of BCAA metabolism may have two-sided effects on aging

BCAAs (Leu, Ile, and Val) are essential amino acids that can only be obtained from the diet, not by endogenous synthesis. We found that valine, leucine, and isoleucine degradation and valine, leucine, and isoleucine biosynthesis were two significant pathways in aging, in which l-Leu and l-Ile were negatively correlated with age. Changes in BCAAs and related pathways in older adults may thus have a two-sided effect on aging.

BCAAs, especially Leu, are powerful stimulants of the mechanistic target of rapamycin (mTOR) pathway [34]. The increase in mTOR activity is considered to be a crucial mechanism of aging [1, 34]. Although accompanied by many negative effects, downregulation of BCAAs can also reduce activation of mTOR signaling and benefit lifespan, which may be an adaptation of organisms to fight aging. On the other hand, a lack of BCAAs may have negative effects on health-span. The decline in relative BCAA content disturbs the amino acid balance in vivo and suppresses appetite, thereby exacerbating age-related malnutrition [35, 36]. Circulating BCAA levels were positively correlated with weight and fat mass in both mice and men [37]. Robust adults tended to consume more BCAAs than frail adults [38]. It is widely believed that BCAAs can stimulate muscle growth as an anabolic signal, and BCAA deficiency may have adverse effects on muscle structure and function in the elderly.

Long-term dietary supplementation of essential amino acids, including BCAAs, may be a powerful method for the prevention and treatment of sarcopenia [39]. However, the effect of pure BCAA supplements in stimulating muscle growth and fighting aging is controversial. Currently, it is widely believed that BCAA supplementation would hardly have benefits to muscle growth, as protein synthesis requires not only signal regulation from BCAAs, but also other signals and abundant amino acid substrates [40]. Similarly, supplementation or restriction of BCAAs hardly has any major effect on aging in the absence of other nutrients [41].

Moreover, we observed up-regulation of l-Val in the old group, without statistical correlation with age, which was different from l-Leu and l-Ile. With similarities in structure and function, the three BCAAs are generally considered to be synchronous. BCAA levels are not only dependent on dietary intake, but are also affected by degradation [41]. Leu is a ketogenic amino acid, Val is a glucogenic amino acid, and Ile is a ketogenic and glucogenic amino acid. The differences in the degradation pathways could explain the unsynchronism of BCAA changes with age to some extent.

### Strengths and limitations

This study aimed to screen age-related biomarkers from serum samples using untargeted metabolomics methods. The operation procedures of clinical data and sample collection, physical function measurement, and metabolomics analysis were all standardized, which ensured the credibility of this research. However, chronological age has limitations in assessing aging and physiological function, without considering the influence of genes and the external environment on aging. In addition, the source of the recruited participants was a single-center and the sample size was relatively small. As a result, the extension of our results is limited to the Chinese population from diverse regions, ethnicities, and cultures. There is a gap in the number of participants from different sexes and a lack of comparison of the metabolic profiles between sexes. Besides, there was a lack of adjustment of interference factors, such as diet and environment in the data analysis. Although these limitations restrict the representation of our results, we initially reveal the metabolic features of aging in China, which provide potential targets for the quantification and intervention of aging. Longitudinal research and verification of aging biomarkers and mechanisms based on multiple populations of each sex will be carried out in the future.

## Conclusions

Our study shows the application of metabolomics platforms in aging research, using this technique to screen 80 candidate biomarkers and two potential pathways associated with aging. We identified that down-regulation of LPs is a significant characteristic of aging in both humans and many animal models, the mechanism of which may be related to the synthesis and function of mitochondrial membranes. Dysfunction of BCAA degradation and biosynthesis probably has a two-sided effect on aging. Further studies need to be performed in a longitudinal cohort of other populations to confirm our observations.

## Supplementary Information

**Additional file 1**: ** Figure S1 **PCA score plot of quality control samples. **Figure S2 **Pairing comparison matrix of 13 QC samples after log10 conversion. Each point in the scatter plot below the diagonal represents a metabolite, and the tight straight line distribution of all points indicates that the data are highly consistent in the two QC samples. The corresponding correlation coefficient is above the diagonal, which is greater than 0.99, indicating good consistency and high data quality. **Figure S3 A)** Scree plot and loading plot of PCA between old and young groups. (a) The Scree plot shows that principal component 1 (Dimention-1: 17.8%) and 2 (Dimention-2: 11%) accounted for nearly 30% of the inter-group differences. (b,c) The loading plots show the first 10 metabolites that make up the first two principal components. **B) **OPLS-DA of metabolites from old and young groups. (a) In random permutation test, the model shows no overfitting phenomenon (pR2Y = 0.05, pQ2 = 0.05). (b) The explainability (R2Y) and predictability (Q2Y) of the model are about 0.5. (c) In the ROC curve, AUC approximately equals to 1 (sensitivity = 0.97, specificity = 1.00). **Table S1 **Detailed information of 66 standard compounds applied for the identification of metabolites. **Table S2** Metabolites summary. We totally identified 349 metabolites of 46 categories from the serum samples of subjects. **Table S3 **Commercial standard information of reagents. **Table S4** Metabolites with VIP > 1, FC > 1.5 or < 1/1.5 and P value < 0.05. **Table S5 **Enriched metabolite sets of aging adults with fold enrichment > 1 and p < 0.05. **Table S6** Over-represented pathways related to aging with raw P < 0.05.

## Data Availability

The data sets used and analyzed during the current study are available from the corresponding author on reasonable request.

## Abbreviations

LC–MS: Liquid chromatography–mass spectrometry
CCr: Creatinine clearance
QC: Quality control
PCA: Principal component analysis
OPLS-DA: Orthogonal partial least squares discriminant analysis
AUC: Area under the receiver operating characteristic curve
MSEA: Metabolite set enrichment analysis
MetPA: Metabolic pathway analysis
UPLC–QTOF: Ultra-performance liquid chromatography–quadrupole time-of-flight
ROC: Receiver operating characteristic
VIP: Variable importance in the projection
FC: Fold change
Val: Valine
Ile: Isoleucine
Leu: Leucine
BCAA: Branched chain amino acid
LP: Lysophospholipid
LPC: Lysophosphatidylcholine
LPE: Lysophosphatidylethanolamine
PL: Phospholipid
PC: Phosphatidylcholine
PE: Phosphatidylethanolamine
mTOR: Mechanistic target of rapamycin
