# Supplementary material for: Lysophospholipids and branched chain amino acids are associated with aging: a metabolomics-based study of Chinese adults
Source: Eur J Med Res. 2023 Feb 2;28:58. doi: 10.1186/s40001-023-01021-w (PMC9893616; doi:10.1186/s40001-023-01021-w)
Supplement: Supplementary file 1 — Additional file 1: Figure S1 PCA score plot of quality control samples. Figure S2 Pairing comparison matrix of 13 QC samples after log10 conversion. Each point in the scatter plot below the diagonal represents a metabolite, and the tight straight line distribution of all points indicates that the data are highly consistent in the two QC samples. The corresponding correlation coefficient is above the diagonal, which is greater than 0.99, indicating good consistency and high data quality. Figure S3 A) Scree plot and loading plot of PCA between old and young groups. (a) The Scree plot shows that principal component 1 (Dimention-1: 17.8%) and 2 (Dimention-2: 11%) accounted for nearly 30% of the inter-group differences. (b,c) The loading plots show the first 10 metabolites that make up the first two principal components. B) OPLS-DA of metabolites from old and young groups. (a) In random permutation test, the model shows no overfitting phenomenon (pR2Y = 0.05, pQ2 = 0.05). (b) The explainability (R2Y) and predictability (Q2Y) of the model are about 0.5. (c) In the ROC curve, AUC approximately equals to 1 (sensitivity = 0.97, specificity = 1.00). Table S1 Detailed information of 66 standard compounds applied for the identification of metabolites. Table S2 Metabolites summary. We totally identified 349 metabolites of 46 categories from the serum samples of subjects. Table S3 Commercial standard information of reagents. Table S4 Metabolites with VIP > 1, FC > 1.5 or < 1/1.5 and P value < 0.05. Table S5 Enriched metabolite sets of aging adults with fold enrichment > 1 and p < 0.05. Table S6 Over-represented pathways related to aging with raw P < 0.05. [file 40001_2023_1021_MOESM1_ESM.docx]

**Additional file 1**

**Figure S1** PCA score plot of quality control samples.

**Figure S2** Pairing comparison matrix of 13 QC samples after log10 conversion. Each point in the scatter plot below the diagonal represents a metabolite, and the tight straight line distribution of all points indicates that the data are highly consistent in the two QC samples. The corresponding correlation coefficient is above the diagonal, which is greater than 0.99, indicating good consistency and high data quality.

**Figure S3 A)** Scree plot and loading plot of PCA between old and young groups. (a) The Scree plot shows that principal component 1 (Dimention-1: 17.8%) and 2 (Dimention-2: 11%) accounted for nearly 30% of the inter-group differences. (b,c) The loading plots show the first 10 metabolites that make up the first two principal components. **B)** OPLS-DA of metabolites from old and young groups. (a) In random permutation test, the model shows no overfitting phenomenon (pR2Y = 0.05, pQ2 = 0.05). (b) The explainability (R2Y) and predictability (Q2Y) of the model are about 0.5. (c) In the ROC curve, AUC approximately equals to 1 (sensitivity = 0.97, specificity = 1.00).

**Table S1** Detailed information of 66 standard compounds applied for the identification of metabolites.

| **Standards** | **Source** | **Identifier** |
| --- | --- | --- |
| 4-Hydroxyproline | Sigma | 56250-5G |
| 5-Hydroxy-L-tryptophan | sigma | H9772-100MG |
| Acetylcholine chloride | Sigma | A6625-25G |
| Adenosine | Sigma | A9251-5G |
| Allantoin | Sigma | 5670-25G |
| Benzoic acid-d5 | Cambridge Isotope Laboratories | DLM-122-1 |
| Betaine-D11 | Cambridge Isotope Laboratories | DLM-407-1 |
| Choline-d13 | Cambridge Isotope Laboratories | DLM-141-0.1 |
| cis-Aconitic acid | Sigma | 122750-25G |
| Citric acid-d4 | Cambridge Isotope Laboratories | DLM-3487-0.5 |
| Creatine-D3 | Cambridge Isotope Laboratories | DLM-1302-0.25 |
| Creatinine-d3 | Cambridge Isotope Laboratories | DLM-3653-0.1 |
| DL-Glutamic acid-d5 | Cambridge Isotope Laboratories | DLM-357-0.25 |
| DL-Isocitric acid trisodium salt hydrate | Sigma | I1252-1G |
| DL-Serine-d3 | Cambridge Isotope Laboratories | DLM-1073-1 |
| Fumaric acid-d4 | Cambridge Isotope Laboratories | DLM-7654-1 |
| Gamma-Aminobutyric acid | Sigma | A5835-10MG |
| Glutaric acid-d4 | Cambridge Isotope Laboratories | DLM-3106-5 |
| Glycerophosphocholine | Macklin | C824554-1g |
| Glycine | Sigma | G7126-100G |
| Guanine | J&K | 223698-25g |
| Hypoxanthine-d3 | Cambridge Isotope Laboratories | DLM-2923-0.1 |
| Indoleacetic acid | J&K | 148807-5g |
| Indolelactic acid | Macklin | I849392-25mg |
| Inosine-15N4 | Cambridge Isotope Laboratories | NLM-4264-0.01 |
| Isoleucine-d10 | Cambridge Isotope Laboratories | DLM-141-0.1 |
| Kynurenic acid-d5 | Cambridge Isotope Laboratories | DLM-7374-PK |
| Kynurenine | Sigma | 61250-250MG |
| L-Alanine-d7 | Cambridge Isotope Laboratories | DLM-251-PK |
| L-Arginine-d7 | Cambridge Isotope Laboratories | DLM-541-0.1 |
| L-Asparagine(13C4) | Cambridge Isotope Laboratories | CLM-8699-H-0.05 |
| L-Aspartic acid-d3 | Cambridge Isotope Laboratories | DLM-546-0.1 |
| L-Citrulline-d4 | Cambridge Isotope Laboratories | DLM-6039-0.01 |
| L-Cystine | Sigma | C8755-100G |
| Leucine-d10 | Cambridge isotopes | DLM-567-0.25 |
| L-Glutamine-d5 | Cambridge Isotope Laboratories | DLM-1826-0.1 |
| L-Histidine-d5 | Cambridge Isotope Laboratories | DLM-7855 |
| L-lactate-d3 | Cambridge Isotope Laboratories | DLM-9071-0.25 |
| L-Lysine-d9 | Cambridge Isotope Laboratories | DLM-570-0.1 |
| L-Methionine-d3 | Cambridge Isotope Laboratories | DLM-431-1 |
| l-ornithine | TCI | O0064-25g |
| L-pipecolic acid | Sigma | P2519-100MG |
| L-Proline-d7 | Cambridge Isotope Laboratories | DLM-487-0.1 |
| L-Threonine-(13C4,15N) | Cambridge Isotope Laboratories | CNLM-587-0.1 |
| L-Tryptophan-d8 | Cambridge Isotope Laboratories | DLM-6903-0.25 |
| L-Tyrosine-D7 | Cambridge Isotope Laboratories | DLM-589-0.05 |
| L-Valine | Sigma | V0500-1G |
| Malic acid-d3 | Cambridge Isotope Laboratories | DLM-9045-0.1 |
| Oxaloacetic acid | Sigma | O4126-5G |
| Oxoglutaric acid | ALDRICH | 75892-25G |
| Oxypurinol | Sigma | 42688-10MG |
| Pantothenic acid | Sigma | 21210-5G-F |
| P-cresol sulfate-d7 | Cambridge Isotope Laboratories | DLM-9786-0.01 |
| Phenylalanine-d8 | Cambridge Isotope Laboratories | DLM-372-1 |
| Phosphorylcholine | Sigma | P0378-5G |
| Pyroglutamic acid | Sigma | P5960-25G |
| Pyruvate-D3 | Cambridge isotopes | DLM-6068-0.5G |
| Sarcosine-d3 | Cambridge Isotope Laboratories | DLM-6874-0.1 |
| Succinic acid-d4 | Cambridge Isotope Laboratories | DLM-584-1 |
| Taurine-13C2 | Cambridge Isotope Laboratories | CLM-6622-0.25 |
| Thymine | J&K | 207930-5g |
| Trimethylamine N-oxide-d9 | Cambridge Isotope Laboratories | DLM-4779-1 |
| Urea-(13C,15N2) | Cambridge Isotope Laboratories | CLM-234-0.5 |
| Uric acid-(13C; 15N3) | Cambridge Isotope Laboratories | CNLM-10617-0.001 |
| Uridine-d2 | Cambridge Isotope Laboratories | DLM-7693-0.05 |
| Xanthine-15N2 | Cambridge Isotope Laboratories | NLM-1698-0.1 |

**Table S2** Metabolites summary. We totally identified 349 metabolites of 46 categories from the serum samples of subjects.

| **Class** | **Metabolite Name** | **Database ID** | **Confidence level** | **m/z_rt** | **Ionization modes** | **MS2 characteristic ions** |
| --- | --- | --- | --- | --- | --- | --- |
| Acyl carnitines | L-Acetylcarnitine | HMDB0000201 | 2 | 204.1233_2.27 | pos | 145.0498, 144.1018, 85.0287, 60.0824 |
|  | L-Palmitoylcarnitine | HMDB0000222 | 2 | 400.3421_10.53 | pos | 341.2678, 239.2355, 144.1004, 85.0282, 60.0809 |
|  | Hexanoylcarnitine | HMDB0000756 | 2 | 260.1859_6.34 | pos | 201.1122, 85.0291 |
|  | Stearoylcarnitine | HMDB0000848 | 2 | 428.3730_11.42 | pos | 369.2994, 85.0278 |
|  | Dodecanoylcarnitine | HMDB0002250 | 2 | 344.2795_9.13 | pos | 285.2084, 184.0774, 144.1078, 118.0902, 85.0291, 60.0831 |
|  | Oleoylcarnitine | HMDB0005065 | 2 | 426.3579_10.75 | pos | 367.2840, 144.1015, 85.0287, 60.0833 |
|  | Tetradecanoylcarnitine | HMDB0005066 | 2 | 372.3104_9.77 | pos | 313.2367, 114.0926, 85.0289, 60.0826, 46.0700 |
|  | Arachidyl carnitine | HMDB0006460 | 2 | 456.4050_11.91 | pos | 397.3318, 210.158, 85.0286 |
|  | Linoelaidyl carnitine | HMDB0006461 | 2 | 424.3421_10.21 | pos | 365.2685, 144.1019, 85.0283, 60.0827 |
|  | Valerylcarnitine | HMDB0013128 | 2 | 246.1701_5.47 | pos | 187.0956, 85.0295, 60.0827 |
|  | Decatrienoylcarnitine | HMDB0013325 | 2 | 310.2012_7.55 | pos | 269.0467, 251.1270, 167.1036, 121.1018, 85.0289, 60.0848 |
|  | trans-2-Tetradecenoylcarnitine | HMDB0013329 | 2 | 370.2951_9.32 | pos | 311.2169, 210.1542, 144.1031, 85.0309, 60.0838 |
|  | O-decanoyl-L-carnitine | HMDB0062631 | 2 | 316.2477_8.46 | pos | 257.1745, 155.1422, 144.1008, 85.0288, 57.0339 |
|  | Isovalerylcarnitine | HMDB0000688 | 2 | 246.1703_5.35 | pos | 187.0958, 85.0291, 60.0837, 57.0738 |
|  | L-Octanoylcarnitine | HMDB0000791 | 2 | 288.2167_7.64 | pos | 229.1425, 144.1025, 127.1101, 85.0291, 60.0831 |
|  | Propionylcarnitine | HMDB0000824 | 2 | 218.1390_3.87 | pos | 159.0644, 85.0292, 57.0341 |
|  | Butyrylcarnitine | HMDB0002013 | 2 | 232.1540_4.72 | pos | 173.0819, 144.1019, 85.0295, 71.0501, 60.0827 |
|  | cis-5-Tetradecenoylcarnitine | HMDB0002014 | 2 | 370.2951_9.41 | pos | 311.2215, 209.1881, 191.1783, 157.0495, 144.1031, 85.0288, |
|  | cis-4-Decenoylcarnitine | HMDB0240585 | 2 | 314.2324_8.28 | pos | 255.1586, 144.1009, 85.0291, 60.0845 |
|  | trans-Hexadec-2-enoyl carnitine | HMDB0006317 | 2 | 398.3266_10.95 | pos | 339.2531, 85.0285 |
|  | 9-Decenoylcarnitine | HMDB0013205 | 2 | 314.2329_8.14 | pos | 255.1585, 153.1281, 144.1007, 135.117485.0286, 60.0845 |
|  | 2-Octenoylcarnitine | HMDB0013324 | 2 | 286.2015_7.01 | pos | 227.1279, 144.1023, 125.0979, 85.0289, 60.0830 |
|  | 3, 5-Tetradecadiencarnitine | HMDB0013331 | 2 | 368.2795_9.09 | pos | 309.2065, 189.1635, 85.0285, 60.0853 |
|  | 9,12-Hexadecadienoylcarnitine | HMDB0013334 | 2 | 396.3105_9.59 | pos | 337.2376, 144.1025, 85.0286 |
|  | 3-hydroxyoctanoyl carnitine | HMDB0061634 | 2 | 304.2117_6.35 | pos | 287.2216, 199.1694, 111.1163, 85.0295, 60.0828 |
|  | Isobutyrylcarnitine | HMDB0000736 | 2 | 232.1544_4.64 | pos | 173.0813, 144.1021, 85.0283, 71.0502, 60.0818, |
|  | isomer of 2-Octenoylcarnitine | — | 3 | 286.2014_7.07 | pos | 227.1272, 144.1028, 125.0985, 85.0300, 60.0829 |
|  | isomer of 3-hydroxyoctanoyl carnitine | — | 3 | 304.2118_6.43 | pos | 287.2212, 199.1699, 111.1169, 85.0301, 60.0826 |
|  | Isomer of decatrienoylcarnitine | — | 3 | 310.2015_7.34 | pos | 251.1278， 167.1042， 121.1015， 85.0291, 60.0841 |
|  | isomer of 3, 5-Tetradecadiencarnitine | — | 3 | 368.2796_9.22 | pos | 309.2062, 85.0281 |
| Alcohols | Pantothenic acid | HMDB0000210 | 1 | 218.1035_4.53 | neg | 146.0820, 88.0403, 71.0518 |
| Amines | Phytosphingosine | HMDB0004610 | 2 | 318.30027_9.38 | pos | 300.2901, 114.0948, 57.0704 |
| Amino acids | 1-Methylhistidine | HMDB0000001 | 2 | 170.0924_1.29 | pos | 152.0819, 135.0555, 124.0872, 109.0963, 107.0608 |
|  | Betaine | HMDB0000043 | 1 | 118.0859_1.41 | pos | 59.0742, 58.0654, 42.0345 |
|  | Creatine | HMDB0000064 | 1 | 132.0764_1.49 | pos | 114.0667, 90.0555, 87.0602, 45.0544 |
|  | Pipecolic acid | HMDB0000070 | 1 | 128.0716_2.20 | neg | 82.0671 |
|  | L-Glutamic acid | HMDB0000148 | 1 | 146.04588_1.41 | neg | 128.0326, 102.0553, 74.0248 |
|  | L-Tyrosine | HMDB0000158 | 1 | 180.0669_3.61 | pos | 165.0508, 147.0435, 136.0752, 123.0444, 119.0488, 95.0493, 91.0545 |
|  | L-Phenylalanine | HMDB0000159 | 1 | 164.0714_4.37 | neg | 147.0443, 103.0552, 91.0548, 72.0095 |
|  | L-Alanine | HMDB0000161 | 1 | 90.0543_1.36 | pos | 44.0501 |
|  | L-Proline | HMDB0000162 | 1 | 116.0706_1.54 | pos | 98.0601, 70.0661 |
|  | L-Threonine | HMDB0000167 | 1 | 118.0509_1.37 | neg | 74.0249, 56.0134 |
|  | L-Asparagine | HMDB0000168 | 1 | 133.0605_1.32 | pos | 116.0352, 87.0556, 74.0241, 46.0287 |
|  | L-Isoleucine | HMDB0000172 | 1 | 130.0873_3.54 | neg | 84.0802 |
|  | L-Histidine | HMDB0000177 | 1 | 156.0762_1.29 | pos | 110.0716, 95.0608, 93.0451, 83.0611, 81.0437, 56.0494 |
|  | L-Lysine | HMDB0000182 | 1 | 147.1126_1.17 | pos | 130.0855, 84.0813, 67.0558, 56.0506, |
|  | L-Serine | HMDB0000187 | 1 | 104.0350_1.32 | neg | 74.0252, 56.0152, 42.0351 |
|  | L-Aspartic acid | HMDB0000191 | 1 | 134.0448_1.32 | pos | 116.0345, 88.0394, 74.0238, 59.0693, 46.0274 |
|  | L-Cystine | HMDB0000192 | 1 | 241.0309_1.30 | pos | 224.0053, 195.0265, 165.9998, 120.0123, 88.0398 |
|  | N6-Acetyl-L-lysine | HMDB0000206 | 2 | 189.1234_2.01 | neg | 169.0981, 145.0982, 127.0874, 101.1082, 58.0296 |
|  | Ornithine | HMDB0000214 | 1 | 133.0971_1.17 | pos | 116.0701, 70.0658 |
|  | Taurine | HMDB0000251 | 1 | 124.0072_1.34 | neg | 79.9571, 63.9614 |
|  | Pyroglutamic acid | HMDB0000267 | 1 | 130.0498_2.66 | pos | 102.0566, 84.0454, 74.0688, 56.0518 |
|  | Urea | HMDB0000294 | 1 | 61.0396_1.44 | pos | 44.0133 |
|  | 5-Hydroxy-L-tryptophan | HMDB0000472 | 1 | 219.0778_3.93 | neg | 201.0676, 175.0893, 157.0767, 132.0449 |
|  | L-Arginine | HMDB0000517 | 1 | 175.1188_1.29 | pos | 158.0909, 130.0965, 116.0704, 88.0761, 72.0818, 70.0659, 60.0565 |
|  | Creatinine | HMDB0000562 | 1 | 114.0660_1.43 | pos | 86.0739, 44.0529 |
|  | L-Glutamine | HMDB0000641 | 1 | 147.0765_1.32 | pos | 130.0490, 102.0554, 84.0452, 56.0506, 41.0394 |
|  | L-Leucine | HMDB0000687 | 1 | 130.0872_3.70 | neg | 113.0601, 88.0406, 84.0819 |
|  | L-Methionine | HMDB0000696 | 1 | 150.0581_2.27 | pos | 133.0315, 104.0526, 102.0547, 87.0266, 74.0592, 61.0115, 56.0502 |
|  | L-Aspartyl-L-phenylalanine | HMDB0000706 | 2 | 279.0985_1.61 | neg | 261.0880, 235.1085, 164.0706, 131.0461, 91.0554 |
|  | L-Homoserine | HMDB0000719 | 2 | 118.05097_1.89 | neg | 100.0401, 72.047 |
|  | 4-Hydroxyproline | HMDB0000725 | 1 | 132.0649_1.37 | pos | 114.0556, 86.0608, 68.0503, 58.0657 |
|  | N-Acetyl-L-alanine | HMDB0000766 | 2 | 130.0505_3.65 | neg | 112.0399, 88.0401, 86.0245, 58.0295 |
|  | L-Valine | HMDB0000883 | 1 | 116.0716_1.97 | neg | 59.0157 |
|  | Citrulline | HMDB0000904 | 1 | 176.1028_1.39 | pos | 159.0758, 113.0705, 70.0663 |
|  | L-Tryptophan | HMDB0000929 | 1 | 203.0822_4.98 | neg | 186.0548, 159.0926, 142.0660, 116.0505, 74.0243 |
|  | DL-2-Aminooctanoic acid | HMDB0000991 | 2 | 158.1186_5.74 | neg | 140.1082, 114.1287, 112.1135, 72.0089 |
|  | N6,N6,N6-Trimethyl-L-lysine | HMDB0001325 | 2 | 189.1598_1.31 | pos | 172.1333, 143.1545, 114.1278, 84.0807 |
|  | Symmetric dimethylarginine | HMDB0001539 | 2 | 203.1503_1.49 | pos | 131.0823, 114.0561, 85.0774, 71.0618 |
|  | Methionine sulfoxide | HMDB0002005 | 2 | 166.0530_1.41 | pos | 102.0549, 74.0235, 56.0493 |
|  | L-Homocysteic acid | HMDB0002205 | 2 | 182.0128_1.65 | neg | 164.0021, 120.9961, 80.9651 |
|  | 5-methoxy-L-tryptophan | HMDB0002339 | 2 | 235.1073_5.4 | pos | 217.0968, 200.0706, 147.0917, 92.0497, 44.0495 |
|  | N2-Acetylornithine | HMDB0003357 | 2 | 173.0929_1.62 | neg | 155.0823, 131.0824. 129.1025, 114.0551 |
|  | 4-Guanidinobutanoic acid | HMDB0003464 | 2 | 146.0920_2.01 | pos | 128.0821, 100.0871, 87.0445, 60.0561 |
|  | N-Acetylglutamine | HMDB0006029 | 2 | 189.0870_1.54 | pos | 171.0762, 160.0591, 143.0819, 130.0497, 102.0547, 88.0391 |
|  | Prolylhydroxyproline | HMDB0006695 | 2 | 229.1180_1.69 | pos | 132.0644, 70.0671 |
|  | Gamma-Aminobutyric acid | HMDB0000112 | 1 | 102.0561_1.43 | neg | 87.0446, 86.0606, 69.034 |
|  | Gamma-Glutamyltyrosine | HMDB0011741 | 2 | 311.1242_4.39 | pos | 248.0916, 182.0808, 165.0542, 136.0761, 84.0445 |
|  | N-Acetyl-L-methionine | HMDB0011745 | 2 | 192.0688_4.31 | pos | 174.0581, 150.0588, 146.0814, 132.0481, 77.0422 |
|  | N-Acetylvaline | HMDB0011757 | 2 | 158.0829_5.26 | neg | 116.0718, 98.0613, 74.0245 |
|  | Glycine | HMDB0000123 | 1 | 76.0389_1.31 | pos | 58.0294 |
|  | Phenylalanylphenylalanine | HMDB0013302 | 2 | 313.1544_6.32 | pos | 267.1495, 166.0865, 120.0812, 103.0556, 93.0702, 91.0545 |
|  | Sarcosine | HMDB0000271 | 1 | 88.0401_1.50 | neg | 42.0345 |
|  | Alanylaspartic acid | HMDB0028683 | 2 | 203.0678_1.44 | neg | 185.0558, 159.0776, 141.0661, 115.0870, 88.0402 |
|  | Alanylleucineine | HMDB0028691 | 2 | 203.1391_3.92 | pos | 132.1016, 114.0911, 86.0965, 44.0494 |
|  | Gamma-Glutamyl Glutamine | HMDB0028833 | 2 | 276.1188_1.62 | pos | 259.0904, 213.0885, 147.0763, 130.0494, 84.0445 |
|  | Leucylalanine | HMDB0028922 | 2 | 203.1390_2.27 | pos | 90.0549, 60.0692, 44.0493 |
|  | Leucyl-Aspartate | HMDB0028925 | 2 | 247.1290_4.20 | pos | 230.1018, 229.1181, 118.0851, 72.0798 |
|  | Leucyl-Glutamate | HMDB0028928 | 2 | 259.1290_5.05 | neg | 241.1169, 223.1067, 197.1270. 130.0869, 128.0341 |
|  | Leucyl-Serine | HMDB0028938 | 2 | 219.1337_1.78 | pos | 131.1177, 86.0965, 69.0695 |
|  | Phenylalanylaspartic acid | HMDB0028991 | 2 | 279.0992_4.59 | neg | 262.0731, 261.0879, 235.1181, 217.0983 |
|  | Phenylalanyltryptophan | HMDB0029006 | 2 | 352.16557_6.37 | pos | 306.1601, 205.0969, 188.0704, 120.0811 |
|  | Tyrosyl-Alanine | HMDB0029098 | 2 | 253.1180_5.38 | pos | 253.1171, 235.1084, 217.0977, 119.0611, 118.0655, 92.0495, 72.0474 |
|  | Tyrosyl-Glutamate | HMDB0029104 | 2 | 309.1099_4.39 | neg | 291.0989, 247.1095, 128.0356, 119.0507 |
|  | N-Methyl-1H-indole-3-propanamide | HMDB0032756 | 2 | 203.1179_9.80 | pos | 175.1223, 142.0651, 132.0677, 120.0437, 92.0503, 77.0386 |
|  | N-gamma-L-Glutamyl-L-methionine | HMDB0034367 | 2 | 277.0862_4.29 | neg | 259.0752, 148.0417, 128.0346, 46.9976 |
|  | Pyroglutamine | HMDB0062558 | 2 | 129.0656_1.46 | pos | 112.0381, 101.0706, 84.0442, 58.0653 |
|  | N-Formyl-L-methionine | HMDB0001015 | 2 | 176.0387_5.11 | neg | 146.0277, 132.0485, 102.0379, 46.9958 |
|  | Alpha-N-Phenylacetyl-L-glutamine | HMDB0006344 | 2 | 263.1042_5.74 | neg | 245.0928, 128.0345, 127.0515 |
|  | L-gamma-glutamyl-L-isoleucine | HMDB0011170 | 2 | 259.1300_4.87 | neg | 241.1168, 223.1065, 197.1270, 130.0870, 128.0350 |
|  | N-acetyltryptophan | HMDB0013713 | 2 | 245.0937_6.85 | neg | 203.0813, 98.0241, 58.0811 |
|  | Valsartan | HMDB0014323 | 2 | 436.2321_9.72 | pos | 418.2238, 408.2289, 352.1771, 306.1712, 291.1482, 235.0979, 207.0901 |
|  | Lenticin | HMDB0061115 | 2 | 247.1438_5.32 | pos | 188.0703, 146.0598, 144.0811, 118.0647, 91.0551, 60.0812 |
|  | Arg Ser Phe | METLIN 15959 | 2 | 407.2055_8.00 | neg | 361.2005, 331.1911, 297.1445 |
|  | Pro Tyr Val Tyr | METLIN 207438 | 2 | 539.2504_7.26 | neg | 521.2408, 333.2128, 99.0176 |
|  | Pro Tyr Tyr Val | METLIN 207476 | 2 | 539.2504_7.26 | neg | 521.2408, 333.2128, 99.0176 |
|  | Ser Phe Val Phe | METLIN 225423 | 2 | 497.2397_8.67 | neg | 451.2354, 113.0722 |
|  | Ile Gly Asp | METLIN 23025 | 2 | 304.1505_7.66 | pos | 171.1135, 86.0958, 44.0491 |
|  | Thr Ile Phe Tyr | METLIN 234378 | 2 | 541.2661_7.08 | neg | 523.2559, 423.1931, 113.0849 |
|  | Thr Ile Tyr Phe | METLIN 234663 | 2 | 541.2663_7.45 | neg | 523.2562, 423.1933, 175.0964, 113.0849 |
|  | Thr Leu Phe Tyr | METLIN 235178 | 2 | 541.2666_6.91 | neg | 495.2613, 483.2618, 89.0246 |
|  | Thr Leu Tyr Phe | METLIN 235463 | 2 | 541.2668_7.22 | neg | 495.2615, 467.2665, 224.1369, 89.0245 |
|  | Thr Tyr Phe Ile | METLIN 239166 | 2 | 541.2662_6.80 | neg | 523.2561, 423.1925, 89.0244 |
|  | Succinyl-leucyl-agmatine | METLIN 414580 | 2 | 344.2277_5.26 | pos | 327.2015, 283.1749, 177.1115, 133.0858, 89.0592 |
|  | Nocardicin C | METLIN 71625 | 2 | 485.1672_6.72 | neg | 309.1331, 175.0235, 113.0260 |
|  | N-Acetyl-L-leucyl-L-proline | METLIN 837905 | 2 | 269.1509_7.06 | neg | 251.1402, 207.1500, 145.0600, 127.0512 |
|  | tert-Butyl-3-amino-1,4,6,7-tetrahydro-5H-pyrazolo[4,3-c]pyridine-5-carboxylate | METLIN 862224 | 2 | 239.1493_4.77 | pos | 221.0969, 133.0796, 89.0594, 45.0330 |
|  | L-2-Aminoadipic acid | PubChem 92136 | 2 | 160.06153_2.96 | neg | 142.0501, 116.0711, 98.0604, 59.0131 |
|  | S-Glutathionyl-L-cysteine | PubChem CID 10455148 | 2 | 425.0797_1.52 | neg | 338.0486, 320.0383, 288.0665, 179.0499, 145.0625 |
| Aminoxides | Trimethylamine N-oxide | HMDB0000925 | 1 | 76.0753_1.40 | pos | 60.0444, 58.0653 |
| Benzene | 4-Hydroxyphenylpyruvic acid | HMDB0000707 | 2 | 269.0927_8.66 | neg | 225.1026, 132.0461, 92.0508 |
|  | 3-Methylphenylacetic acid | HMDB0002222 | 2 | 149.0611_8.36 | neg | 105.0709, 79.0551 |
|  | Phenacemide | HMDB0015253 | 2 | 179.0814_9.74 | pos | 91.0541, 61.0388, 42.0295 |
|  | p-Toluenesulfonic acid | HMDB0059933 | 2 | 171.0122_5.19 | neg | 144.9967, 104.9655, 80.9651 |
| Benzoic acids | Hippuric acid | HMDB0000714 | 2 | 178.0511_5.88 | neg | 134.0604, 132.0451, 77.0402 |
|  | Benzoic acid | HMDB0001870 | 1 | 121.0295_7.51 | neg | 93.0344, 77.0401 |
|  | 2,4-dihydroxy-3-methoxybenzoic acid | HMDB0125542 | 2 | 183.0301_5.78 | neg | 165.0194, 164.0117, 139.0402 |
|  | 2,5-dihydroxy-4-methoxybenzoic acid | HMDB0130482 | 2 | 183.0299_6.59 | neg | 139.0406, 99.0095, 83.0135, 81.0364 |
|  | Salicylic acid | HMDB0001895 | 2 | 137.0242_7.79 | neg | 93.0343, 44.9979 |
|  | Syringic acid | HMDB0002085 | 2 | 197.0451_5.73 | neg | 181.0139, 167.0346, 153.0555, 137.0237 |
|  | 3-Hydroxyhippuric acid | HMDB0006116 | 2 | 194.0451_5.08 | neg | 150.0558, 93.0348, |
|  | 2,6-Dihydroxybenzoic acid | HMDB0013676 | 2 | 153.0194_6.11 | neg | 109.0298, 81.0347 |
|  | 4-Hydroxyhippuric acid | HMDB0013678 | 2 | 194.0451_4.82 | neg | 150.0558, 93.0347 |
| Bile acids | Glycocholic acid | HMDB0000138 | 2 | 464.3015_8.65 | neg | 402.3090, 353.2454, 74.0245 |
|  | Allochenodeoxycholic acid | HMDB0000514 | 2 | 391.2842_9.86 | neg | 373.2747, 355.2642, 355.2640, 81.0344 |
|  | Chenodeoxycholic acid | HMDB0000518 | 2 | 391.2855_10.99 | neg | 373.2749, 345.2797 |
|  | Cholic acid | HMDB0000619 | 2 | 407.2807_9.55 | neg | 389.2691, 345.2795, 83.0495, 59.0129 |
|  | Deoxycholic acid | HMDB0000626 | 2 | 391.2853_11.22 | neg | 373.2748, 345.2798 |
|  | Deoxycholic acid glycine conjugate | HMDB0000631 | 2 | 448.3069_9.09 | neg | 430.2962, 347.2951, 74.0246, 56.0143 |
|  | Glycoursodeoxycholic acid | HMDB0000708 | 2 | 448.3076_9.56 | neg | 404.3178, 402.2972, 386.3084, 384.2936, 74.0273 |
|  | Murocholic acid | HMDB0000811 | 2 | 391.2858_9.53 | neg | 373.2749, 287.2019 |
|  | Taurodeoxycholic acid | HMDB0000896 | 2 | 498.2893_9.29 | neg | 452.3348, 74.0258 |
|  | Ursodeoxycholic acid | HMDB0000946 | 2 | 391.2856_9.72 | neg | 345.2795 |
|  | Chenodeoxyglycocholic acid | HMDB0006898 | 2 | 448.3069_8.72 | neg | 404.3175, 386.3101, 355.2714, 74.0260 |
|  | 12a-Hydroxy-3-oxocholadienic acid | HMDB0000385 | 2 | 385.2387_9.45 | neg | 367.2281, 349.2161, 341.2489 |
|  | Glycohyocholic acid | HMDB0240607 | 2 | 464.3015_8.31 | neg | 402.3090, 345.2798, 74.0245, 56.0141 |
|  | Glycochenodeoxycholate-3-sulfate | HMDB0002497 | 2 | 528.2643_8.85 | neg | 448.3401, 446.3078, 303.2341, 74.0259 |
|  | Chenodeoxycholic acid 3-sulfate | HMDB0002586 | 2 | 471.2425_8.67 | neg | 453.2315, 425.2369, 96.9603 |
| Carbohydrates | D-Glucuronic acid | HMDB0000127 | 2 | 193.0355_1.37 | neg | 175.0247, 147.0302, 131.0353, 119.0355, 59.0142 |
|  | Glyceric acid | HMDB0000139 | 2 | 105.01933_1.61 | neg | 87.0082, 75.0082, 72.9926, 59.0133, 43.0184 |
|  | Gluconic acid | HMDB0000625 | 2 | 195.0514_1.43 | neg | 177.0419, 129.0187, 99.0083, 75.0091 |
|  | N-Acetylgalactosamine 6-sulfate | HMDB0000841 | 2 | 300.0392_4.32 | neg | 130.0507, 104.0401, 96.9605, 58.0293 |
|  | L-Threonic Acid | HMDB0000943 | 2 | 135.0298_1.47 | neg | 117.0195, 89.0245, 75.0089, 59.0141, |
|  | Phlorin | HMDB0035589 | 2 | 289.0921_1.40 | pos | 271.0818, 253.0697, 127.0385, 85.0289 |
|  | Arabinonic acid | HMDB0000539 | 2 | 165.0405_1.43 | neg | 89.0241, 75.0084, 72.9929, 59.0137, 44.9979 |
|  | N-Acetylmuramate | HMDB0060493 | 2 | 292.1041_9.65 | neg | 248.1146, 200.0569, 155.0618, 131.0609 |
|  | Muramic acid | HMDB0003254 | 2 | 250.0927_1.41 | neg | 89.0241, 71.0137 |
|  | Acetaminophen glucuronide | HMDB0010316 | 2 | 326.0881_4.14 | neg | 175.0235, 150.0556, 113.0241 |
|  | 1-(beta-D-Ribofuranosyl)-1,4-dihydronicotinamide | HMDB0011648 | 2 | 257.1140_3.90 | pos | 240.0850, 125.0698, 108.0451, 89.0612 |
|  | D-Fructofuranose 1,2':2,3'-dianhydride | KEGG C04420 | 2 | 323.0981_1.41 | neg | 305.08781, 119.9851 |
|  | N-Acetylgalactosamine | METLIN 313462 | 2 | 220.0829_1.49 | neg | 202.0724, 166.0847 |
|  | 7-Hydroxyterpineol 8-glucoside | HMDB0033019 | 2 | 331.1760_8.47 | neg | 313.1659, 271.1548, 169.1237, 75.0091 |
| Carboxylic acids | cis-Aconitic acid | HMDB0000072 | 1 | 173.00916_3.85 | neg | 129.0188, 111.0182, 85.0290, 59.0133 |
|  | Citric acid | HMDB0000094 | 1 | 191.0203_2.52 | neg | 173.0084, 154.9969, 129.0191, 111.0090, 87.0092, 85.0299, 57.0353 |
|  | Glyoxylic acid | HMDB0000119 | 2 | 163.0510_7.37 | neg | 119.0595, 92.0491, 45.0054 |
|  | Fumaric acid | HMDB0000134 | 1 | 115.0034_3.19 | neg | 71.0140, 53.0032 |
|  | Isocitric acid | HMDB0000193 | 1 | 191.0201_1.83 | neg | 173.0086, 147.0292, 129.0183, 117.0184, 103.0392, 99.0078, 71.0132 |
|  | Succinic acid | HMDB0000254 | 1 | 117.0192_3.43 | neg | 99.0085, 73.0292, 55.0187 |
|  | Glutaric acid | HMDB0000661 | 1 | 131.0349_4.23 | neg | 87.0547, 69.0365 |
|  | 3,3'-Thiobispropanoic acid | HMDB0031162 | 2 | 177.0225_4.36 | neg | 61.0118, 58.9965 |
| Fatty acids | Oleic acid | HMDB0000207 | 2 | 281.2489_14.37 | neg | 45.3242 |
|  | Palmitic acid | HMDB0000220 | 2 | 255.2333_14.26 | neg | 237.2209, 211.2429, 209.2271 |
|  | Linoleic acid | HMDB0000673 | 2 | 279.2335_13.57 | neg | 261.2225 |
|  | Hydroxyisocaproic acid | HMDB0000746 | 2 | 131.0711_5.81 | neg | 113.0609, 85.0661, 69.0349, 67.0554 |
|  | Octadecanedioic acid | HMDB0000782 | 2 | 313.2389_13.21 | neg | 295.2282, 277.2177, 251.2385 |
|  | Arachidonic acid | HMDB0001043 | 2 | 303.2322_13.36 | neg | 259.2424, 231.2115, 205.1955, 59.0136 |
|  | 2-Oxo-4-methylthiobutanoic acid | HMDB0001553 | 2 | 147.0121_2.21 | neg | 99.0061, 46.9971 |
|  | 2-Hydroxycaproic acid | HMDB0001624 | 2 | 131.07137_6.28 | neg | 113.0610, 85.0666, 69.0352, 44.9994 |
|  | Docosapentaenoic acid (22n-6) | HMDB0001976 | 2 | 329.2483_13.42 | neg | 285.2596, 237.0379, 59.0139 |
|  | 2-Hydroxy-2-methylbutyric acid | HMDB0001987 | 2 | 117.0555_5.13 | neg | 99.0449, 71.0500, 44.9985 |
|  | Itaconic acid | HMDB0002092 | 2 | 129.0193_3.19 | neg | 111.0081, 85.0287, 67.0181 |
|  | Heptadecanoic acid | HMDB0002259 | 2 | 269.2483_14.93 | neg | 251.2371, 59.0132 |
|  | Palmitoleic acid | HMDB0003229 | 2 | 253.2177_13.41 | neg | 235.2065, 209.2271, 59.0135 |
|  | 9,10-DHOME | HMDB0004704 | 2 | 313.2389_13.57 | neg | 295.2277, 277.2171, 251.2382, 59.0138 |
|  | Docosapentaenoic acid (22n-3) | HMDB0006528 | 2 | 329.2483_13.42 | neg | 285.2596, 237.0379, 59.0139 |
|  | 3-Oxododecanoic acid | HMDB0010727 | 2 | 213.1500_10.05 | neg | 195.1392, 59.0148, 41.0052 |
|  | 12-Methyltridecanoic acid | HMDB0031072 | 2 | 227.2022_13.29 | neg | 209.1913, 137.1339 |
|  | 3-Hydroxytetradecanedioic acid | HMDB0000394 | 2 | 273.1703_12.65 | neg | 255.1601, 237.1499, 59.0141 |
|  | 9Z-Heptadecenoic acid | HMDB0062437 | 2 | 267.2327_13.95 | neg | 249.2227, 223.2425， 59.0141 |
|  | Hydroxyoctanoic acid | HMDB0000711 | 2 | 159.1025_8.41 | neg | 141.0923, 113.0977 |
|  | Alpha-Linolenic acid | HMDB0001388 | 2 | 277.2180_13.07 | neg | 259.2061, 233.2272, 59.0136 |
|  | Docosahexaenoic acid | HMDB0002183 | 2 | 327.2322_13.16 | neg | 283.2436, 237.0445, 229.1959, 59.0137 |
|  | 3-carboxy-4-methyl-5-pentyl-2-furanpropanoic acid | HMDB0061643 | 2 | 267.1242_9.93 | neg | 223.1335, 205.1229, 179.1439, 177.1278, 69.0349 |
|  | 2-methyl-tridecanedioic acid | LMFA01170015 | 2 | 257.1755_9.73 | neg | 239.1645, 195.1749, 175.1255 |
|  | 13(S)-HpOTrE | METLIN 36052 | 2 | 309.2079_11.03 | neg | 291.1968, 273.1865, 247.1998, 209.1159, |
| Fatty Acyls | 13-HODE | HMDB0004667 | 2 | 295.2284_11.88 | neg | 277.2189, 195.1394, 183.1392, |
| Flavonoid glycosides | [2-({4,5-dihydroxy-2-[4-(7-hydroxy-4-oxo-3,4-dihydro-2H-1-benzopyran-2-yl)phenoxy]-6-(hydroxymethyl)oxan-3-yl}oxy)-4-hydroxy-4-(hydroxymethyl)oxolan-3-yl]oxidanesulfonic acid | HMDB0124937 | 2 | 629.1177_6.63 | neg | 611.1079, 595.1344 |
| Glycerophosphocholines | Glycerophosphocholine | HMDB0000086 | 1 | 258.1099_1.35 | pos | 184.0729, 166.0626, 124.9993, 104.1074, 86.0970 |
|  | LysoPC(14:0) | HMDB0010379 | 2 | 468.3080_10.33 | pos | 450.2986, 184.0728, 124.9991, 104.1070 |
|  | LysoPC (22:4) | HMDB0010401 | 2 | 572.3702_12.15 | pos | 554.3610, 184.0735, 104.1081 |
|  | LysoPC(18:1) | HMDB0002815 | 2 | 522.3555_12.06 | pos | 504.3451, 445.2699, 258.1110, 184.0735, 124.9989, 104.1076, 86.0981, 60.0839 |
|  | LysoPC(16:0) | HMDB0010382 | 2 | 496.3397_11.69 | pos | 478.3262, 313.2715, 258.1072, 184.0715, 124.9985, 104.1065, 86.0968 |
|  | LysoPC(16:1) | HMDB0010383 | 2 | 494.3239_10.70 | pos | 476.3146, 311.2474, 184.0737, 124.9985, 86.0991104.1076, |
|  | LysoPC(18:2) | HMDB0010386 | 2 | 520.3397_11.15 | pos | 502.3288, 184.0725, 124.9998, 104.1072, 86.0971, 60.0828 |
|  | LysoPC(18:3) | HMDB0010387 | 2 | 518.3238_10.61 | pos | 500.3136, 184.0701, 104.1071 |
|  | LysoPC(20:4) | HMDB0010396 | 2 | 544.3398_11.17 | pos | 526.3287, 258.1104, 184.0733, 124.9989, 104.1072, 86.0968 |
|  | LysoPC(20:5) | HMDB0010397 | 2 | 542.3238_10.48 | pos | 524.3115, 258.1049, 184.0703, 104.1066, 86.0968 |
|  | LysoPC(22:5) | HMDB0010402 | 2 | 570.3558_11.45 | pos | 552.3448, 496.3391, 387.2891, 184.0727, 104.1074 |
|  | LysoPC(22:6) | HMDB0010404 | 2 | 568.3389_11.09 | pos | 550.3285, 258.1105, 184.0734, 134.9990, 104.1075, 86.0973 |
|  | PC (16:0) | METLIN40386 | 2 | 482.3603_12.14 | pos | 341.3112, 283.1776, 184.0701, 104.1074 |
|  | sn2 LysoPC(14:0) | — | 3 | 468.3080_10.09 | pos | 450.2985, 184.0732, 86.0961 |
|  | sn2 LysoPC(16:0) | — | 3 | 496.3399_11.43 | pos | 478.3262, 313.2719, 258.1072, 184.0715, 124.9985, 104.1065, 86.0968 |
|  | sn2 LysoPC(18:3) | — | 3 | 518.3237_10.44 | pos | 500.3136, 184.0701, 104.1071 |
|  | sn2 LysoPC(18:2) | — | 3 | 520.3396_10.93 | pos | 502.3291, 184.0729, 124.9986, 104.1072, 60.0831 |
|  | sn2 LysoPC(18:1) | — | 3 | 522.3552_11.82 | pos | 504.3446, 339.2938, 258.1113, 184.0732, 124.9992, 104.1078, 86.0975 |
|  | sn3 LysoPC(20:5) | — | 3 | 542.3238_10.31 | pos | 524.3110, 184.0731, 104.1069 |
|  | sn2 LysoPC(20:4) | — | 3 | 544.3398_10.96 | pos | 526.3275, 258.1111, 184.0733, 124.9981, 104.1073, 86.0965 |
|  | sn2 LysoPC(22:6) | — | 3 | 568.3389_10.92 | pos | 550.3279, 184.0728, 104.1076 |
|  | sn2 LysoPC(22:5) | — | 3 | 570.3554_11.25 | pos | 552.3449, 496.3395, 387.2893, 184.0725, 104.1073 86.0975 |
|  | sn2 LysoPC (22:4) | — | 3 | 572.3701_12.15 | pos | 554.3610, 184.0736 104.1082 |
| Glycerophosphoethanolamines | LysoPE(16:0) | HMDB0011473 | 2 | 452.2783_11.61 | neg | 255.2326, 196.0390, 152.9959, 140.0121, 78.9599 |
|  | LysoPE(20:1) | HMDB0011484 | 2 | 504.3080_11.59 | pos | 486.2981, 443.2560, 424.3002, 363.2871, 289.2520, 62.0603, 44.0496 |
|  | LysoPE(20:4) | HMDB0011487 | 2 | 502.2928_11.03 | pos | 484.2825, 441.2401, 367.2731, 287.2372, 62.0601, 44.0497 |
|  | LysoPE(20:5) | HMDB0011489 | 2 | 500.2771_10.45 | pos | 482.2667, 359.2588, 142.0267 |
|  | LysoPE(18:1) | HMDB0011505 | 2 | 480.3085_11.95 | pos | 462.2975, 419.2560, 339.2889, 265.2511, 155.0101, 44.0499 |
|  | LysoPE(18:2) | HMDB0011507 | 2 | 476.2783_11.06 | neg | 279.2330, 214.0481, 196.0355, 152.9961, 78.9592 |
|  | LysoPE(22:6) | HMDB0011526 | 2 | 526.293_11.17 | pos | 508.2829, 385.2739, 311.2362, 62.0605, 44.0499 |
|  | sn2 LysoPE(16:0) | — | 3 | 452.2781_11.33 | neg | 255.2324, 196.0388, 140.0122 |
|  | sn2 LysoPE(18:2) | — | 3 | 476.2783_11.06 | neg | 279.2332, 214.0483, 196.0353 |
|  | sn2 LysoPE(18:1) | — | 3 | 480.3082_11.72 | pos | 339.2882, 265.2513, 155.0103 |
|  | sn2 LysoPE(20:4) | — | 3 | 502.2928_10.93 | pos | 484.2825, 441.2401, 367.2749, 287.2385, 62.0601, 44.0497 |
|  | sn2 LysoPE(22:6) | — | 3 | 526.2928_10.88 | pos | 508.2821, 385.2745, 62.0601 |
| Glycerophosphoglycerols | 1-Stearoylglycerophosphoglycerol | HMDB0061697 | 2 | 511.3009_9.59 | neg | 437.2671, 419.2572, 283.2644 |
| Glycosides | Phenylgalactoside | METLIN 65793 | 2 | 255.0883_6.63 | neg | 211.0975, 167.1075, 123.0439, 119.0498, 109.0669, 107.0505, 93.0354, 86.0392 |
| Hydroxy acids | 2-Hydroxybutyric acid | HMDB0000008 | 2 | 131.07137_6.38 | neg | 113.0607, 85.0666, 69.0352, 44.9992 |
|  | Glycolic acid | HMDB0000115 | 2 | 75.0084_1.49 | neg | 56.9976, 44.9979 |
|  | L-Malic acid | HMDB0000156 | 1 | 133.0144_1.87 | neg | 115.0030, 89.0237, 87.0081, 72.9929, 71.0135, 59.0131, 43.0186 |
|  | L-Lactic acid | HMDB0000190 | 1 | 89.0242_2.28 | neg | 71.0141, 43.0188 |
|  | (R)-3-Hydroxybutyric acid | HMDB0000011 | 2 | 103.0400_3.96 | neg | 85.0291, 59.0153, 57.0349 |
|  | 3-Hydroxydodecanedioic acid | HMDB0000413 | 2 | 245.1291_11.2 | neg | 227.1292, 201.1498, 59.0139 |
|  | 2-Hydroxyglutarate | HMDB0059655 | 2 | 147.0300_3.44 | neg | 129.0187, 103.0401, 85.0298, 57.0347 |
|  | 3-Hydroxycapric acid | HMDB0002203 | 2 | 187.1343_9.77 | neg | 169.1231, 141.1287 |
|  | 2,4-Dihydroxybutanoic acid | HMDB0000360 | 2 | 119.0349_2.20 | neg | 75.0088, 57.0345, 43.0191 |
| Imidazoles | Allantoin | HMDB0000462 | 1 | 157.0363_1.49 | neg | 140.0094, 114.0302, 97.0038, 71.0244, 59.0243 |
| Indolecarboxylic acids | Indole-3-carboxylic acid | HMDB0003320 | 2 | 160.0407_7.07 | neg | 142.0295, 116.0501, 90.0341 |
| Indoles | Indoleacrylic acid | HMDB0000734 | 2 | 186.0566_5.35 | neg | 142.0652, 116.0503, |
|  | 5-Hydroxyindoleacetic acid | HMDB0000763 | 2 | 190.0511_4.35 | neg | 146.0616， 144.0463，128.0512 |
|  | 3-(Dimethylaminomethyl)indole | HMDB0035762 | 2 | 175.1230_9.8 | neg | 159.0921, 132.0811, 104.0492, |
| Indolyl carboxylic acids | Indoleacetic acid | HMDB0000197 | 1 | 174.0561_7.57 | neg | 130.0660, 128.0508, 59.0131 |
|  | Indolelactic acid | HMDB0000671 | 1 | 204.0669_6.89 | neg | 186.056, 158.0603, 142.0660, 116.0504, 72.9971 |
|  | Tryptophan 2-C-mannoside | HMDB0240296 | 2 | 365.1353_4.24 | neg | 347.1255, 321.1462, 245.0926, 158.0605, 130.0668, |
| Keto acids | 2-Ketobutyric acid | HMDB0000005 | 2 | 191.0832_7.88 | neg | 147.0926, 98.0249, 92.0503, 59.0155 |
|  | alpha-Ketoisovaleric acid | HMDB0000019 | 2 | 205.0986_9.42 | neg | 177.1029, 92.0518 |
|  | Oxoglutaric acid | HMDB0000208 | 1 | 235.0725_7.89 | neg | 191.0828, 147.0927, 98.0256, 92.0516 |
|  | Oxalacetic acid | HMDB0000223 | 1 | 221.0569_7.95 | neg | 177.0681, 133.0787, 92.051 |
|  | Oxoadipic acid | HMDB0000225 | 2 | 249.0885_8.01 | neg | 205.0982 |
|  | Pyruvic acid | HMDB0000243 | 1 | 177.0672_8.19 | neg | 133.0769, 117.0461, 91.0437, 92.0512 |
|  | Ketoleucine | HMDB0000695 | 2 | 219.1143_9.80 | neg | 175.1234, 117.0457, 92.0497, 91.042 |
|  | 3-Sulfinylpyruvic acid | HMDB0001405 | 2 | 241.0289_6.45 | neg | 197.0397, 153.0499, 107.0484 |
|  | 2-Keto-glutaramic acid | HMDB0001552 | 2 | 234.0885_7.82 | neg | 190.0955, 90.0501 |
|  | 2-Oxoarginine | HMDB0004225 | 2 | 262.1316_6.11 | neg | 218.1415, 92.0502 |
|  | 3-Oxohexanoic acid | HMDB0010717 | 2 | 129.0557_5.73 | neg | 93.0344, 69.0347, 59.0141 |
|  | Alpha-Ketooctanoic acid | HMDB0013211 | 2 | 247.1450_11.01 | neg | 203.1524, 92.0495 |
|  | 4-Hydroxy-2-oxobutanoic acid | HMDB0031204 | 2 | 207.0773_8.42 | neg | 163.0871, 92.0501 |
|  | xi-3-Hydroxy-2-oxobutanoic acid | HMDB0039324 | 2 | 207.0775_7.28 | neg | 163.0869, 92.0503 |
|  | 3-Oxodecanoic acid | HMDB0010724 | 2 | 185.1187_9.28 | neg | 167.1077, 139.1129, 45.0001 |
| Morphinans | Dihydroisomorphine-6-glucuronide | HMDB0061137 | 2 | 462.1764_7.38 | neg | 418.1875, 285.1201, 253.1371, 185.0250, 175.0241 |
| Nucleosides | Pseudouridine | HMDB0000767 | 2 | 243.0625_2.19 | neg | 225.0513, 213.0515, 183.0409, 153.0302, 111.0191, 41.9982 |
|  | 3'-AMP | HMDB0003540 | 2 | 346.0560_2.19 | neg | 328.0449, 134.0467, 96.9691, 78.9602 |
| Organic phosphoric acids | Glycerophosphoglycerol | HMDB0240316 | 2 | 245.0437_1.45 | neg | 213.0172, 168.9908, 152.9961, 78.9593 |
|  | Sphingosine 1-phosphate (d16:1-P) | HMDB0060061 | 2 | 350.2107_9.22 | neg | 78.9595 |
| Organic sulfuric acids | p-Cresol sulfate | HMDB0011635 | 1 | 187.0077_6.51 | neg | 107.0502, 80.9649, 79.9571 |
|  | Homovanillic acid sulfate | HMDB0011719 | 2 | 261.0070_3.96 | neg | 217.0177, 96.9604 |
|  | 2-methoxyacetaminophen sulfate | HMDB0062550 | 2 | 260.0239_3.74 | neg | 215.9975, 201.9813, 178.0511, 96.9603, 80.9655 |
|  | Indoxyl sulfate | HMDB0000682 | 2 | 212.0028_5.86 | neg | 132.0449, 80.9648, 79.9574 |
|  | 2-hydroxy-3-(sulfooxy)benzoic acid | HMDB0134105 | 2 | 232.9754_2.29 | neg | 188.9861, 109.0296, 80.9654 |
|  | Hydroquinone sulfate | HMDB0240263 | 2 | 188.9866_5.31 | neg | 109.0293, 80.9651 |
|  | 4-Methylcatechol 1-sulfate | HMDB0240459 | 2 | 203.0013_5.61 | neg | 96.9603, 80.9655 |
|  | 4-Methylcatechol 2-sulfate | HMDB0240461 | 2 | 203.0011_6.40 | neg | 187.0059, 177.0219, 159.0113, 125.0599 |
|  | 3-Methylcatechol 2-sulfate | HMDB0240663 | 2 | 203.0019_5.61 | neg | 175.0068, 134.9577, 89.0035, 80.9655, 65.0399 |
|  | Dihydrocaffeic acid 3-sulfate | HMDB0041721 | 2 | 261.0071_3.59 | neg | 242.9968, 215.0018, 96.9602 |
|  | Pyrocatechol sulfate | HMDB0059724 | 2 | 188.9868_5.24 | neg | 160.9915, 96.9605, 80.9655, |
|  | Paracetamol sulfate | HMDB0059911 | 2 | 230.0130_4.54 | neg | 203.9975, 188.0026, 134.9764, 120.9605 |
|  | Phenol sulphate | HMDB0060015 | 2 | 172.9910_5.53 | neg | 93.0343 |
|  | 4-vinylphenol Sulfate | HMDB0062775 | 2 | 199.0073_7.19 | neg | 119.0506, 96.0605 |
| Organooxygen | L-Kynurenine | HMDB0000684 | 1 | 209.0919_4.34 | pos | 192.0646, 174.0544, 146.0600, 94.0656, 74.0256 |
| Phenol ethers | [3-(4-methoxyphenyl) propoxy]sulfonic acid | HMDB0135750 | 2 | 245.0492_7.59 | neg | 165.0929, 164.0848, 80.9649, 79.9568 |
| Phenols | p-Hydroxyphenylacetic acid | HMDB0000020 | 2 | 151.0400_7.47 | neg | 133.0292, 107.0451, 105.0342 |
|  | 2-Methoxyhydroquinone | METLIN 263518 | 2 | 139.0397_6.60 | neg | 109.0181, 93.0368, 81.0343, 67.0213 |
| Phenylpropanoic acids | D-Phenyllactic acid | HMDB0000563 | 2 | 165.0559_6.70 | neg | 103.0558, 44.9987 |
|  | Hydroxyphenyllactic acid | HMDB0000755 | 2 | 181.0501_5.13 | neg | 163.0396, 135.0443, 119.0497, 107.0498, 93.0342, 72.9931 |
|  | 3-(2-hydroxyphenyl)butanoic acid | HMDB0141019 | 2 | 179.0717_9.24 | neg | 162.0688, 134.0752, |
|  | 3-(2,3,4-trimethoxyphenyl) propanoic acid | HMDB0142074 | 2 | 239.0933_8.87 | neg | 239.0929, 331.0813, 195.1012, 151.1127, |
| Phenylpyruvic acid | Phenylpyruvic acid | HMDB0000205 | 2 | 253.1081_8.98 | neg | 235.0955, 209.1198, 191.1068, 173.0962 |
| Phosphosphingolipids | Sphingosine 1-phosphate | HMDB0000277 | 2 | 378.2410_10.19 | neg | 78.9595 |
| Purine nucleosides | Adenosine | HMDB0000050 | 1 | 266.08948_3.67 | neg | 134.0411, 107.0309, 92.0203 |
|  | Inosine | HMDB0000195 | 1 | 267.0727_3.85 | neg | 135.0309, 108.0200, 92.0241 |
|  | 1-Methylguanosine | HMDB0001563 | 2 | 298.1145_4.13 | pos | 166.0723, 135.0317 |
|  | 8-Hydroxy-deoxyguanosine | HMDB0003333 | 2 | 282.0844_3.78 | neg | 236.0791, 166.0374, 138.0424 |
|  | 2-Methylguanosine | HMDB0005862 | 2 | 296.1006_4.10 | neg | 222.0631, 164.0581, 133.0159, 89.0247 |
|  | 1-Methyladenosine | HMDB0003331 | 2 | 282.1190_2.29 | pos | 150.0765, 133.0507 |
|  | Succinyladenosine | HMDB0000912 | 2 | 384.1149_4.37 | pos | 252.0715, 206.0679, 148.0601, 136.0623 |
| Purines | Guanine | HMDB0000132 | 1 | 152.05669_3.84 | pos | 135.0307, 110.0354, 109.0514, 107.0358, 93.0089, 82.0405, 55.0296 |
|  | Hypoxanthine | HMDB0000157 | 1 | 137.0455_2.42 | pos | 120.0189, 119.0353, 110.0347, 94.0202, 92.0250, 82.0407, 55.0301 |
|  | Uric acid | HMDB0000289 | 1 | 167.0209_2.32 | neg | 124.0153, 97.0041, 96.0204, 69.0093, 41.9985 |
|  | Xanthine | HMDB0000292 | 1 | 151.0263_3.07 | neg | 108.0199, 80.0253, 65.9988, 59.0137, 41.9981 |
|  | Oxypurinol | HMDB0000786 | 1 | 151.0264_3.64 | neg | 133.0151, 181.0185, 80.0250, 41.9992 |
|  | Ethenodeoxyadenosine | HMDB0001786 | 2 | 276.1085_4.61 | pos | 216.0881, 160.0619, 133.0511 |
|  | 9-Methyluric acid | HMDB0001973 | 2 | 181.0362_4.05 | neg | 138.0299, 110.0352, 83.0246 |
|  | 1-Methylguanine | HMDB0003282 | 2 | 166.0719_2.38 | pos | 139.0615, 109.0508, 95.0242, 82.0402 |
|  | Allopurinol | HMDB0014581 | 2 | 137.0456_3.82 | pos | 120.0237, 119.0355, 110.0358, 94.0399, 91.0589, |
|  | Theophylline | HMDB0001889 | 2 | 181.0723_4.86 | pos | 124.0479, 109.0244, 96.0539, 69.0431, 42.0321 |
|  | Theobromine | HMDB0002825 | 2 | 181.0722_4.51 | pos | 163.0619, 138.0655, 110.0718, 83.0599, 54.0322 |
|  | 1-Methyluric acid | HMDB0003099 | 2 | 183.0517_4.04 | pos | 155.0571, 98.0347, |
| Pyridines | 4-Pyridoxic acid | HMDB0000017 | 2 | 182.0459_3.98 | neg | 138.0558, 123.0328, 108.0459, 92.0519 |
|  | Niacinamide | HMDB0001406 | 2 | 123.0553_2.31 | pos | 96.0456, 80.0501, 78.0342, 53.0387, |
|  | 3-Pyridylacetic acid | HMDB0001538 | 2 | 138.0545_1.49 | pos | 120.0442, 92.0496, 65.0389 |
|  | Nornicotine | HMDB0001126 | 2 | 149.1072_9.57 | pos | 132.0809, 122.0968, 108.0811, 65.0377 |
|  | N1-Methyl-2-pyridone-5-carboxamide | HMDB0004193 | 2 | 153.0653_3.88 | pos | 136.0396, 135.0551, 110.0603, 108.0441, 80.0492 |
| Pyrimidine nucleosides | Uridine | HMDB0000296 | 1 | 243.0628_3.55 | neg | 200.0565, 182.0429, 153.0306, 152.0349, 122.0223, 111.0198, 110.0242, 41.9983 |
|  | Ribothymidine | HMDB0000884 | 2 | 257.0775_4.03 | neg | 239.0759, 214.0717, 125.0353, 124.0409, |
| Pyrimidines | Thymine | HMDB0000262 | 1 | 127.0498_4.00 | pos | 107.0244, 85.0037, 55.0183 |
|  | Dihydrouracil | HMDB0000076 | 2 | 113.0348_1.32 | neg | 70.0295, 41.9984 |
| Pyrrolopyrazines | 4-Methylpyrrolo[1,2-a]pyrazine | HMDB0033173 | 2 | 133.0756_8.22 | pos | 92.0487, 63.0225, 51.0224 |
| Quaternary ammonium salts | L-Carnitine | HMDB0000062 | 2 | 162.1122_1.38 | pos | 103.0391, 102.0915, 85.0288, 60.0814, 43.0191 |
|  | Choline | HMDB0000097 | 1 | 104.1066_1.32 | pos | 60.0821, 59.0739, 58.0662, 45.0341, 44.0499 |
|  | Acetylcholine | HMDB0000895 | 1 | 146.1174_1.50 | pos | 87.0445, 60.0816, 43.0184 |
|  | Phosphorylcholine | HMDB0001565 | 1 | 184.0735_1.33 | pos | 124.9998, 98.9842, 88.1122, 84.0806, 60.0809 |
| Quinoline carboxylic acids | Kynurenic acid | HMDB0000715 | 1 | 188.0352_5.19 | neg | 170.0245, 144.0451 |
| Steroidal glycosides | (3a,5b,7a)-23-Carboxy-7-hydroxy-24-norcholan-3-yl-b-D-Glucopyranosiduronic acid | HMDB0002430 | 2 | 567.3171_8.52 | neg | 549.3069, 505.3175, 391.2849, 373.2748, 193.0355, 147.0301 |
|  | Glycochenodeoxycholic acid 3-glucuronide | HMDB0002579 | 2 | 624.3409_8.39 | neg | 448.3075, 175.0241, 113.0241 |
|  | Deoxycholic acid 3-glucuronide | HMDB0002596 | 2 | 567.3182_9.17 | neg | 549.3071, 391.2853, 193.0354, 175.0249, 147.0299, 103.0035 |
|  | Androsterone glucuronide | HMDB0002829 | 2 | 465.2494_8.92 | neg | 421.2595, 405.2285, 289.2179, 193.0351, 147.0298 |
|  | Etiocholanolone glucuronide | HMDB0004484 | 2 | 465.2494_8.78 | neg | 447.2389, 289.2176, 193.0355, 147.0299 |
|  | Pregnanediol-3-glucuronide | HMDB0010318 | 2 | 495.2972_9.09 | neg | 477.2852, 377.2694, 317.2488, 75.0092 |
|  | Fusicoccin H | METLIN 67422 | 2 | 481.2813_9.82 | neg | 463.2705, 363.2543, 283.2658, 113.0621 |
|  | isomer of Fusicoccin H | — | 3 | 481.2808_9.70 | neg | 463.2701, 363.2547, 283.2658, 113.0628 |
|  | Isomer of glycochenodeoxycholic acid 3-glucuronide | — | 3 | 624.3400_8.52 | neg | 448.3081, 175.0243, 113.0239 |
| Steroids | Cortisol | HMDB0000063 | 2 | 363.2160_8.00 | pos | 345.2061, 327.1955, 309.1826, 121.0655 |
|  | (25S)-11alpha,20,26-trihydroxyecdysone | METLIN 57610 | 2 | 511.2909_8.53 | neg | 493.2801, 475.2707, 113.0299, 85.0341 |
| Sulfamic acid | Cyclamic acid | HMDB0031340 | 2 | 178.0544_5.27 | neg | 160.0432, 97.9915, 95.9758, 80.9643 |
| Sulfated steroids | 16alpha-Hydroxy DHEA 3-sulfate | HMDB0062544 | 2 | 383.1540_7.51 | neg | 96.9601 |
|  | Pregnenolone sulfate | HMDB0000774 | 2 | 395.1889_10.60 | neg | 377.1793, 353.1790, 96.9600 |
|  | 5alpha-Pregnan-3beta,20beta-diol 20-sulfate | HMDB0240580 | 2 | 399.2207_9.55 | neg | 96.9605 |
|  | Pregnanolone sulfate | HMDB0240590 | 2 | 397.2049_9.28 | neg | 352.2078, 96.9598 |
|  | Testosterone sulfate | HMDB0002833 | 2 | 367.1591_10.13 | neg | 96.9601 |
| Vitamin | Biotin | HMDB0000030 | 2 | 245.09499_8.45 | pos | 185.0631, 141.0125, 97.0405, 83.0249 |
|  | 23S,25,26-Trihydroxyvitamin D3 | HMDB0060134 | 2 | 431.3177_10.30 | neg | 385.3115, 357.2800, 341.2485, 89.0606 |

Abbreviation: m/z, mass to charge ratio; rt, retetion time.

**Table S3** Commercial standard information of reagents.

| **Standards** | **Source** | **Identifier** |
| --- | --- | --- |
| Pantothenic acid | Sigma | 21210-5G-F |
| Betaine-D11 | Cambridge Isotope Laboratories | DLM-407-1 |
| Creatine-D3 | Cambridge Isotope Laboratories | DLM-1302-0.25 |
| L-pipecolic acid | Sigma | P2519-100MG |
| DL-Glutamic acid-d5 | Cambridge Isotope Laboratories | DLM-357-0.25 |
| L-Tyrosine-D7 | Cambridge Isotope Laboratories | DLM-589-0.05 |
| Phenylalanine-d8 | Cambridge Isotope Laboratories | DLM-372-1 |
| L-Alanine-d7 | Cambridge Isotope Laboratories | DLM-251-PK |
| L-Proline-d7 | Cambridge Isotope Laboratories | DLM-487-0.1 |
| L-Threonine-(13C4,15N) | Cambridge Isotope Laboratories | CNLM-587-0.1 |
| L-Asparagine(13C4) | Cambridge Isotope Laboratories | CLM-8699-H-0.05 |
| Isoleucine-d10 | Cambridge Isotope Laboratories | DLM-141-0.1 |
| L-Histidine-d5 | Cambridge Isotope Laboratories | DLM-7855 |
| L-Lysine-d9 | Cambridge Isotope Laboratories | DLM-570-0.1 |
| DL-Serine-d3 | Cambridge Isotope Laboratories | DLM-1073-1 |
| L-Aspartic acid-d3 | Cambridge Isotope Laboratories | DLM-546-0.1 |
| L-Cystine | Sigma | C8755-100G |
| l-ornithine | TCI | O0064-25g |
| Taurine-13C2 | Cambridge Isotope Laboratories | CLM-6622-0.25 |
| Pyroglutamic acid | Sigma | P5960-25G |
| Urea-(13C,15N2) | Cambridge Isotope Laboratories | CLM-234-0.5 |
| 5-Hydroxy-L-tryptophan | Sigma | H9772-100MG |
| L-Arginine-d7 | Cambridge Isotope Laboratories | DLM-541-0.1 |
| Creatinine-d3 | Cambridge Isotope Laboratories | DLM-3653-0.1 |
| L-Glutamine-d5 | Cambridge Isotope Laboratories | DLM-1826-0.1 |
| Leucine-d10 | Cambridge isotopes | DLM-567-0.25 |
| L-Methionine-d3 | Cambridge Isotope Laboratories | DLM-431-1 |
| 4-Hydroxyproline | sigma | 56250-5G |
| L-Valine | Sigma | V0500-1G |
| L-Citrulline-d4 | Cambridge Isotope Laboratories | DLM-6039-0.01 |
| L-Tryptophan-d8 | Cambridge Isotope Laboratories | DLM-6903-0.25 |
| Gamma-Aminobutyric acid | Sigma | A5835-10MG |
| Glycine | Sigma | G7126-100G |
| Sarcosine-d3 | Cambridge Isotope Laboratories | DLM-6874-0.1 |
| Trimethylamine N-oxide-d9 | Cambridge Isotope Laboratories | DLM-4779-1 |
| Benzoic acid-d5 | Cambridge Isotope Laboratories | DLM-122-1 |
| cis-Aconitic acid | Sigma | 122750-25G |
| Citric acid-d4 | Cambridge Isotope Laboratories | DLM-3487-0.5 |
| Fumaric acid-d4 | Cambridge Isotope Laboratories | DLM-7654-1 |
| DL-Isocitric acid trisodium salt hydrate | Sigma | I1252-1G |
| Succinic acid-d4 | Cambridge Isotope Laboratories | DLM-584-1 |
| Glutaric acid-d4 | Cambridge Isotope Laboratories | DLM-3106-5 |
| Glycerophosphocholine | Macklin | C824554-1g |
| Malic acid-d3 | Cambridge Isotope Laboratories | DLM-9045-0.1 |
| L-lactate-d3 | Cambridge Isotope Laboratories | DLM-9071-0.25 |
| Allantoin | Sigma | 5670-25G |
| Indoleacetic acid | J&K | 148807-5g |
| Indolelactic acid | Macklin | I849392-25mg |
| Oxoglutaric acid | ALDRICH | 75892-25G |
| Oxaloacetic acid | Sigma | O4126-5G |
| Pyruvate-D3 | Cambridge isotopes | DLM-6068-0.5G |
| P-cresol sulfate-d7 | Cambridge Isotope Laboratories | DLM-9786-0.01 |
| Kynurenine | Sigma | 61250-250MG |
| Adenosine | Sigma | A9251-5G |
| Inosine-15N4 | Cambridge Isotope Laboratories | NLM-4264-0.01 |
| Guanine | J&K | 223698-25g |
| Hypoxanthine-d3 | Cambridge Isotope Laboratories | DLM-2923-0.1 |
| Uric acid-(13C; 15N3) | Cambridge Isotope Laboratories | CNLM-10617-0.001 |
| Xanthine-15N2 | Cambridge Isotope Laboratories | NLM-1698-0.1 |
| Oxypurinol | Sigma | 42688-10MG |
| Uridine-d2 | Cambridge Isotope Laboratories | DLM-7693-0.05 |
| Thymine | J&K | 207930-5g |
| Choline-d13 | Cambridge Isotope Laboratories | DLM-141-0.1 |
| Acetylcholine chloride | Sigma | A6625-25G |
| Phosphorylcholine | Sigma | P0378-5G |
| Kynurenic acid-d5 | Cambridge Isotope Laboratories | Cat#DLM-7374-PK |

**Table S4** Metabolites with VIP > 1, FC > 1.5 or < 1/1.5 and P value < 0.05.

| **Metabolite Name** | **Class** | **Database ID** | **VIP** | **P value** | **FOLD: old/young** |
| --- | --- | --- | --- | --- | --- |
| 5-methoxy-L-tryptophan | Amino acids | HMDB0002339 | 1.256646103 | 0.008 | 0.622179514 |
| Tyrosyl-Alanine | Amino acids | HMDB0029098 | 1.303662647 | 0.006 | 0.497115636 |
| N-Methyl-1H-indole-3-propanamide | Amino acids | HMDB0032756 | 1.994633242 | <0.001 | 0.630116141 |
| Alpha-N-Phenylacetyl-L-glutamine | Amino acids | HMDB0006344 | 1.390936442 | 0.006 | 1.932438749 |
| Glyoxylic acid | Carboxylic acids | HMDB0000119 | 2.571687666 | <0.001 | 0.651531729 |
| Isocitric acid | Carboxylic acids | HMDB0000193 | 2.626122612 | <0.001 | 1.529028545 |
| 3-Hydroxytetradecanedioic acid | Fatty acids | HMDB0000394 | 1.261760959 | 0.005 | 0.539949799 |
| 13-HODE | Fatty Acyls | HMDB0004667 | 2.049146487 | 0.003 | 1.547536753 |
| LysoPC (22:4) | Glycerophosphocholines | HMDB0010401 | 1.876860942 | <0.001 | 0.525239269 |
| LysoPE(20:1) | Glycerophosphoethanolamines | HMDB0011484 | 2.140394039 | <0.001 | 0.511020879 |
| LysoPE(20:4) | Glycerophosphoethanolamines | HMDB0011487 | 2.889879766 | <0.001 | 0.263941308 |
| LysoPE(20:5) | Glycerophosphoethanolamines | HMDB0011489 | 1.653207934 | 0.001 | 0.476109509 |
| LysoPE(18:2) | Glycerophosphoethanolamines | HMDB0011507 | 2.086282532 | <0.001 | 0.548672585 |
| LysoPE(22:6) | Glycerophosphoethanolamines | HMDB0011526 | 2.543749659 | <0.001 | 0.378016258 |
| sn2 LysoPE(20:4) | Glycerophosphoethanolamines |  | 2.693876325 | <0.001 | 0.545597185 |
| sn2 LysoPE(22:6) | Glycerophosphoethanolamines |  | 2.112087812 | <0.001 | 0.612783519 |
| Indoleacrylic acid | Indoles | HMDB0000734 | 1.088616878 | 0.018 | 0.60042997 |
| 3-(Dimethylaminomethyl)indole | Indoles | HMDB0035762 | 2.01085994 | <0.001 | 0.612522207 |
| 2-Ketobutyric acid | Keto acids | HMDB0000005 | 1.602915698 | 0.013 | 0.654747765 |
| alpha-Ketoisovaleric acid | Keto acids | HMDB0000019 | 2.19039093 | <0.001 | 0.582004853 |
| xi-3-Hydroxy-2-oxobutanoic acid | Keto acids | HMDB0039324 | 1.596627196 | 0.005 | 0.662953059 |
| Dihydroisomorphine-6-glucuronide | Morphinans | HMDB0061137 | 1.659275379 | 0.004 | 0.532770023 |
| Glycerophosphoglycerol | Organic phosphoric acids | HMDB0240316 | 1.408864901 | 0.044 | 1.742661412 |
| p-Cresol sulfate | Organic sulfuric acids | HMDB0011635 | 1.216026572 | 0.017 | 1.847817543 |
| 4-Methylcatechol 2-sulfate | Organic sulfuric acids | HMDB0240461 | 1.341071638 | 0.015 | 2.141905727 |
| Pyrocatechol sulfate | Organic sulfuric acids | HMDB0059724 | 1.231889406 | 0.027 | 1.691051245 |
| Allopurinol | Purines | HMDB0014581 | 1.343185981 | 0.032 | 2.293937572 |
| 3-Pyridylacetic acid | Pyridines | HMDB0001538 | 2.915573766 | <0.001 | 0.246248859 |
| Androsterone glucuronide | Steroidal glycosides | HMDB0002829 | 1.266694953 | 0.005 | 0.627274356 |
| Testosterone sulfate | Sulfated steroids | HMDB0002833 | 1.780665885 | <0.001 | 0.579791934 |

Abbreviation: VIP, variable importance in the projection; FC, fold change.

**Table S5** Enriched metabolite sets of aging adults with fold enrichment > 1 and p < 0.05.

| **Metabolite Set** | **Match status** | **Fold enrichment** | **FDR** | **Total** | **Metabolites** | | **Raw *p*** |
| --- | --- | --- | --- | --- | --- | --- | --- |
|  |  |  |  |  | **Up-regulated** | **Down-regulated** |  |
| Valine, Leucine and Isoleucine Degradation | 5/9 | 2.16 | 1 | Alpha-ketoisovaleric acid, Biotin, L-Glutamic acid, L-Isoleucine, Oxoglutaric acid, Succinic acid, L-Leucine, Ketoleucine, L-Valine | L-Valine | Alpha-ketoisovaleric acid, L-Isoleucine, L-Leucine, Ketoleucine | 0.0469 |

**Table S6** Over-represented pathways related to aging with raw P < 0.05.

| **KEGG ID** | **Pathway** | **Match status** | **Metabolites** | | **Raw p** | **FDR** | **Impact** |
| --- | --- | --- | --- | --- | --- | --- | --- |
|  |  |  | **Up-regulated** | **Down-regulated** |  |  |  |
| hsa00280 | Valine, leucine and isoleucine degradation | 5/5 | L-Valine (C00183) | 3-Methyl-2-oxobutanoic acid (C00141), L-Isoleucine (C00407), 4-Methyl-2-oxopentanoate (C00233), L-Leucine (C00123) | 0.002 | 0.128 | 0.022 |
| hsa00290 | Valine, leucine and isoleucine biosynthesis | 6/7 | L-Valine (C00183) | L-Leucine (C00123), 3-Methyl-2-oxobutanoic acid (C00141), 2-Oxobutanoate (C00109), L-Isoleucine (C00407), 4-Methyl-2-oxopentanoate (C00233) | 0.003 | 0.128 | 0.000 |

**Figure S1**


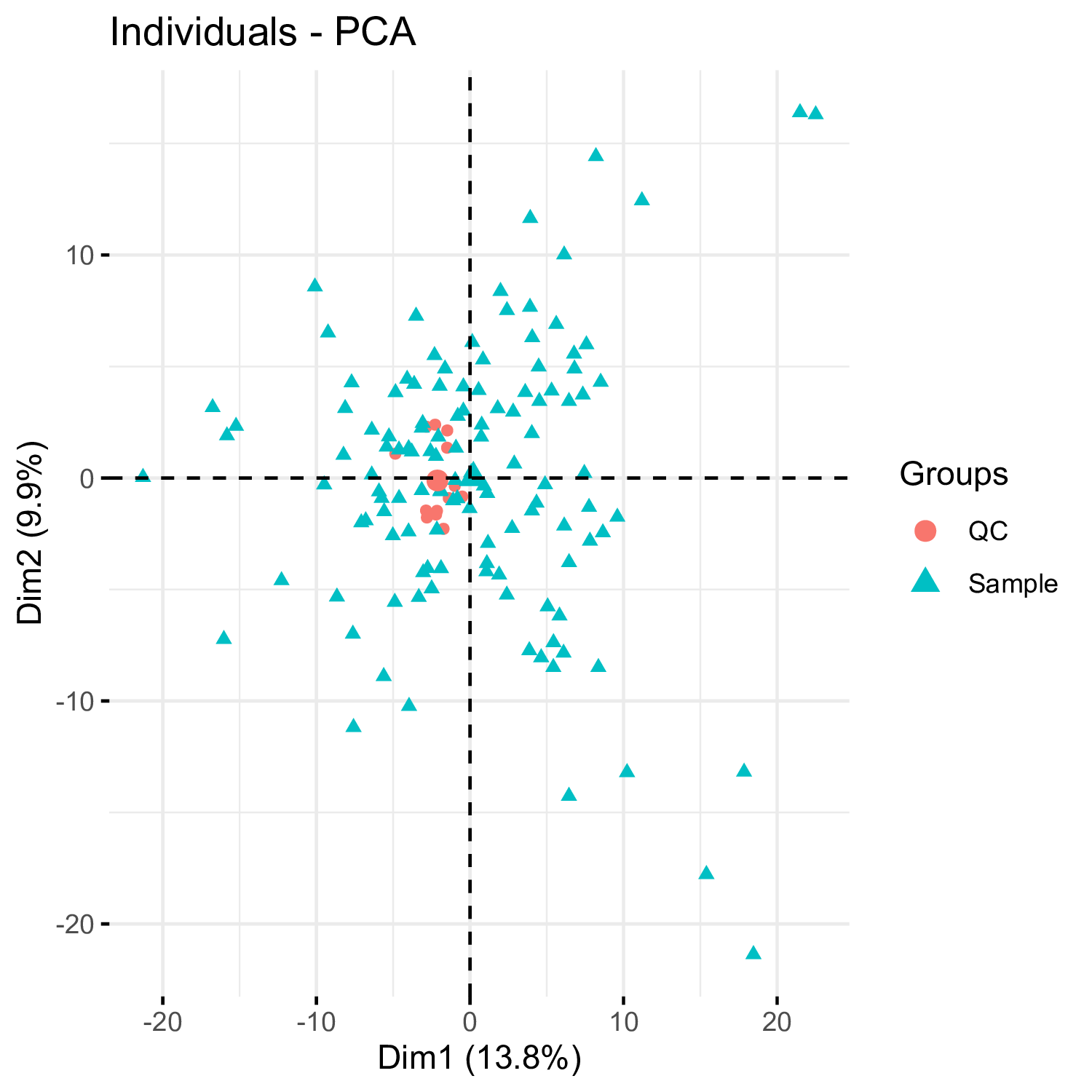


**Figure S2**

**
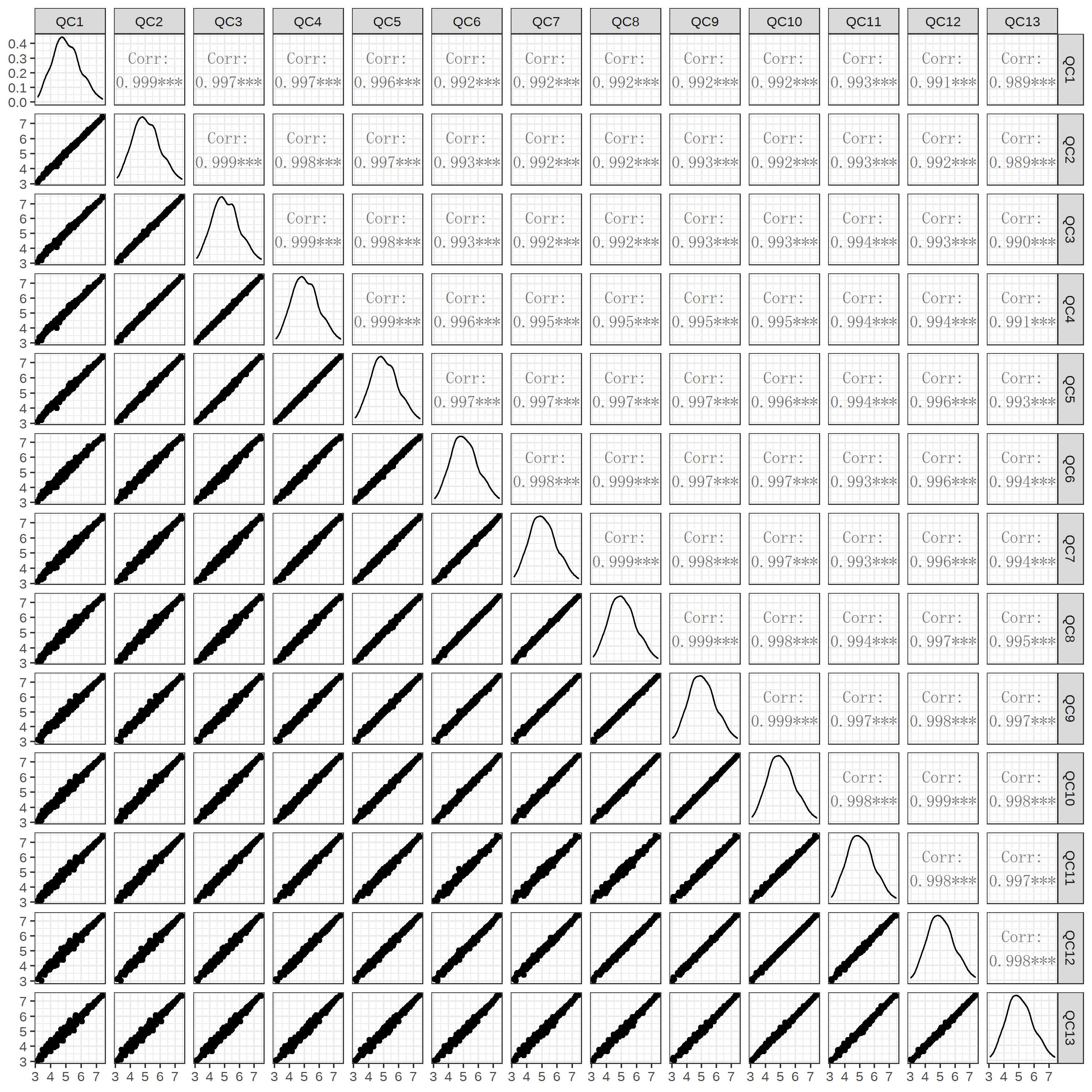
**

**Figure S3**

**
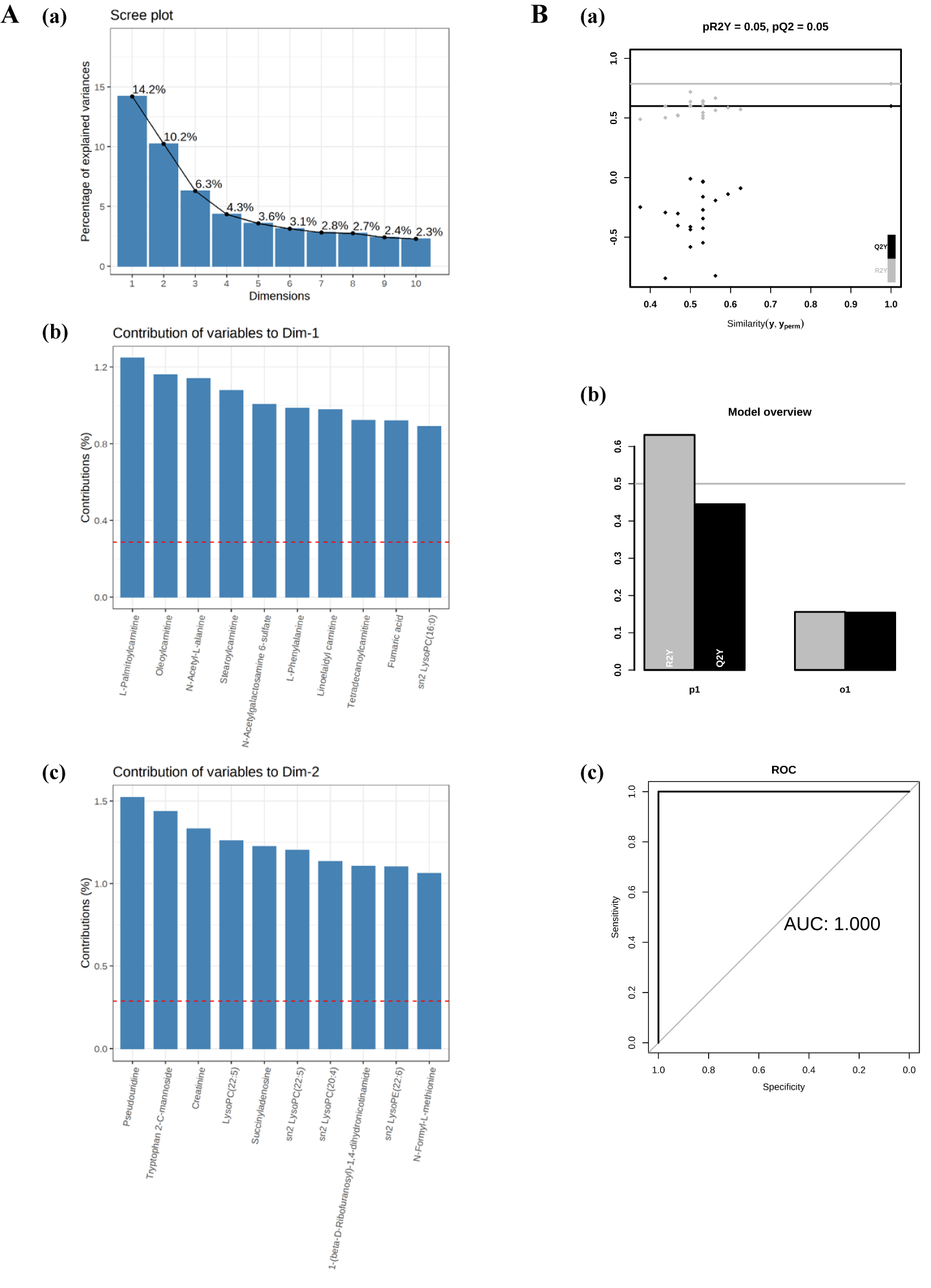
**
